# Delay discounting, probability discounting, and interdental cleaning frequency

**DOI:** 10.1186/s12903-022-02328-6

**Published:** 2022-07-29

**Authors:** Anthony DeFulio, Mark Rzeszutek

**Affiliations:** 1 Department of Psychology, Western Michigan University, 1903 W Michigan Ave., Mail Stop 5439, Kalamazoo, MI 49008 USA; 2 Present Address: University of Kentucky College of Medicine, Lexington, USA

**Keywords:** Behavioral economics, Flossing, Oral health, Preventive health behavior, Impulsivity

## Abstract

**Background:**

Interdental cleaning is recommended by dentists but many people do not floss regularly. The health benefits of interdental cleaning are delayed, and sensitivity to delay is an important factor in many health behaviors. Thus, the present studies explore the relationship between frequency of flossing, and sensitivity to delayed and probabilistic outcomes.

**Method:**

Crowd-sourced subjects were recruited in two studies (n = 584 and n = 321, respectively). In both studies, subjects reported their frequency of flossing and completed delay discounting and probability discounting tasks. Discounting was measured with area under the curve, and linear regression was used to analyze the results.

**Results:**

Findings show that higher levels of delay discounting were associated with less frequent flossing (*p* < 0.001, both studies). In contrast, probability discounting was not significantly associated with flossing frequency (*ns*, both studies).

**Conclusion:**

The findings are consistent with prior studies involving other health behaviors such as attendance at primary care and medication adherence. Results suggest that interventions that reduce delay discounting may help promote regular interdental cleaning, and that delay discounting is a more robust predictor of health behaviors than probability discounting. In addition, interdental cleaning appears to be a reasonable target behavior for evaluating potentially generalizable behavioral health interventions. Thus, interventions that are successful in promoting oral health behaviors should be considered as candidates for evaluation in other health behavior domains.

**Supplementary Information:**

The online version contains supplementary material available at 10.1186/s12903-022-02328-6.

## Background

The evidence in support of the oral health benefits of interdental cleaning (e.g., flossing) is limited [1, 2]. Nevertheless, dentists recommend it to their patients as part of a basic oral health care routine, and a public-facing webpage maintained by the American Dental Association characterizes interdental cleaning as an “essential part of taking care of your teeth and gums” (ADA website).

An estimated 32% of Americans report engaging in daily interdental cleaning [3]. For most people, the decision to floss is likely based on the recommendations of dental health professionals, or interactions with family, friends, or other trusted sources. This perspective is supported by the findings that the interdental cleaning behavior of mothers is the strongest predictor of the interdental cleaning behavior of their children [4], and that normative support is a key factor in the development of flossing automaticity [5]. It is also consistent with studies showing that flossing is positively correlated with expectations regarding its positive health impact [6]. Overall, belief that interdental cleaning is good appears to arise from social interactions and is common among people who regularly floss. Relatedly, a patient’s level of trust with their health care provider is associated with whether they follow medical advice [7].

Another important commonality among people who regularly floss is that they are confident they can do it properly, which is described as self-efficacy [8, 9]. Conversely, people who view flossing as an unpleasant activity are especially unlikely to do it [8–10]. Such barriers include concerns about bleeding gums and pain caused by flossing. Overall, the key predictors related to interdental cleaning are simple. The decision to integrate interdental cleaning into an oral health routine appears to be largely determined by a combination of an expectation it will be beneficial, confidence in the ability to do it, and a lack of barriers such as pain.

Many interventions have successfully targeted these factors as a means of increasing interdental cleaning (see [11], for a review). Although these interventions are informed by a variety of theoretical frameworks, they share similar features and collectively suggest that individualized assistance with planning is an especially effective intervention strategy. Integrating goal setting and self-monitoring into these individualized plans is also recommended [11].

Importantly, interdental cleaning shares a fundamental similarity to most other preventive health behaviors. Specifically, the health benefits are delayed instead of immediate, and are typically realized over timeframes from weeks to decades. The effects of these delays on health behavior have been studied extensively, resulting in the development of broadly applicable models of delay discounting [12, 13]. Individual differences in sensitivity to delayed outcomes is independent of other personality factors [14]. Integrating the study of probability discounting into studies of delay discounting enhances the value of the findings and involves a common analytical approach [15]. As such, investigation of the roles of delay and probability discounting in interdental cleaning may allow for further tailoring of interventions designed to promote interdental cleaning. In addition, comparison of interdental cleaning to other health behaviors may facilitate understanding of common behavioral processes that underlie preventive health behaviors. Thus, the purpose of the present study was to investigate associations between delay and probability discounting and self-reported frequency of interdental cleaning. Two experiments were conducted. As an exploratory measure, message framing was manipulated in the second experiment. Framing can affect attitudes, beliefs, and behavior regarding health decisions [16], as well as discounting [17]. Thus, inclusion of this variable in Experiment 2 allowed for additional points of comparison with the health behavior and decision-making literature.

## Methods

### Subjects

Subjects were workers recruited from Amazon Mechanical Turk (MTurk). MTurk is a crowdsourcing marketplace that allows for the electronic recruitment of convenience samples for academic research [18]. In both experiments, potential subjects were required to (1) have at least 100 approved human interface tasks (HITs), (2) have at least a 95% HIT approval rate, (3) be located the in US, and (4) not have already completed either experiment previously. This last requirement ensured that the experiments consisted of wholly unique samples. Based on Rzeszutek et al. [19] subjects were required to complete a short set of percentage comprehension questions as a screener in advance of taking the surveys. An incorrect answer on any screener question resulted in exclusion from both studies. The first page of the survey was a consent document that described the study and specified that continuation of participation served as demonstration of consent. In Experiment 1, compensation was $0.25 for completion of the screener and $3.00 for completion of the experimental survey. In Experiment 2, no compensation was provided for the screener, and $3.50 was paid for completion of the experimental survey. Sample size for the larger experiments from which these data were collected was determined based on an detecting a small to medium effect size (*r* = 0.2) for other covariates.

### Procedures

Surveys were hosted by the online platform Qualtrics. In both experiments, demographic information and flossing were assessed in identical fashion prior to the discounting survey. All surveys used in the study are available as supplementary files. Flossing was assessed with a single multiple-choice question in which subjects selected how often they flossed. There were six options for flossing frequency: At least once a day, at least once a week, at least once a month, at least once in 6 months, at least once a year, and less than once a year. Subjects responded to the monetary discounting questions by moving sliders (i.e., dragging a button on a line to indicate a percentage or preference towards one of two outcomes). For each question, a smaller monetary amount (i.e., $500) was shown on the left and a larger monetary amount (i.e., $1000) was shown on the right. Probabilities were always expressed as percentages to subjects.

**Fig. 1:**
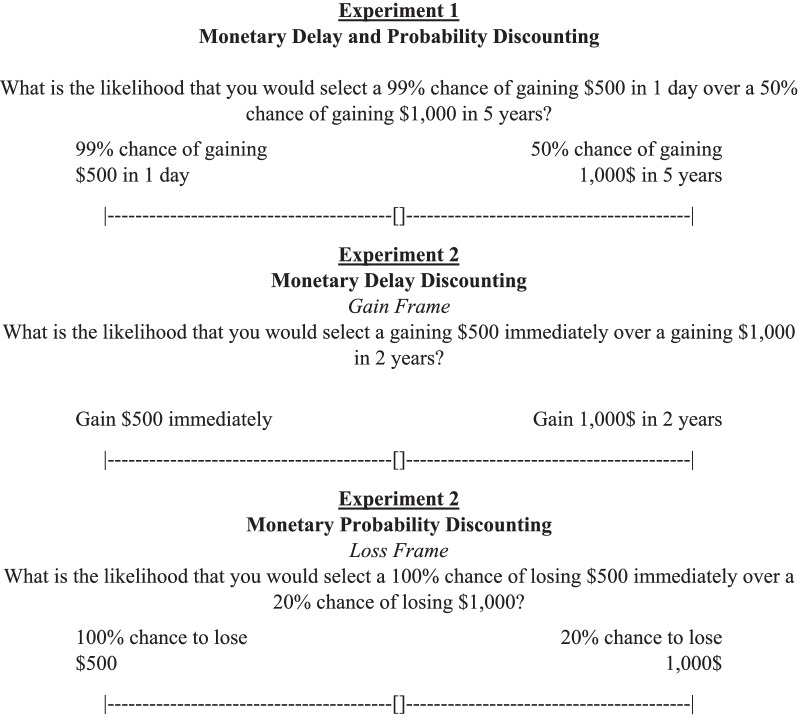
Example schematics of question layout for monetary discounting. []: default position of the slider. Top panel: monetary discounting question that combined both delay and probability from Experiment 1. Middle panel: monetary discounting question that presented a choice between monetary gains that change by delay from Experiment 2. Bottom panel: monetary discounting question that presented a choice between monetary losses that change by probability from Experiment 2

Figure 1 shows how the questions were presented, and complete instructions and experimental arrangements are provided separately (Additional files 1, 2). In Experiment 1, the smaller outcome was always set at the smallest delay (i.e., 1 day) and largest probability (i.e., 0.99). The larger outcome varied across questions in terms of probability and delay. Sets of five questions were presented in order of descending probability (0.99, 0.8, 0.5, 0.2, and 0.0). Within the set, delay to the larger outcome was held constant. Across sets, delay to the larger outcome was varied (1 day, 1 month, 6 months, 2 years, and 5 years). This resulted in 25 combinations of delays and probabilities of the larger outcome. Experiment 2 had similar questions, but instead of questions consisting of both delay and probability, outcomes were only delayed or probabilistic. The delays used were the same as in Experiment 1, except that 2 years was used as the largest delay instead of 5 years. Probabilities were the same as Experiment 1. Experiment 2 replicated Experiment 1, but differed in how probability and delay were manipulated. Experiment 2 also included an additional dichotomous independent variable; the framing of the values in terms of gain or loss. That is, some questions consisted of choices between smaller sooner/certain gains and larger later/uncertain gains, while others featured smaller sooner/certain losses and larger later/uncertain losses. This resulted in four different conditions, delayed gains, delayed losses, probabilistic gains, and probabilistic losses. Each condition was presented in a single block of five questions, with either ascending delays or descending probabilities, for a total of 20 questions.

### Data analysis

Discounting was measured using area under the curve with ordinal scaling (AUC_ord_; [20]). This was calculated with the formula AUC_ord_ = ∑_*i*_(1/*n*_*x*_)[(*y*_1_ + *y*_2_)/2], where *n*_*x*_ is the number of total intervals between data points, or the number of questions in a condition minus 1, and where *y*_1_ and *y*_2_ are the normalized responses of the two points that indicate an interval. Because subject responses were already in percentages, where 0% was sliding all the way the left, and 100% was all the way to the right, normalization was simply the response divided by 100 to produce a proportion. The area of the polygon is calculated for each interval and then summed up to create a total value between 0 and 1. Because each interval is equally spaced, area differences between contiguous data points in delays or probabilities are carry equal weight for purposes of analysis. Higher values indicate lower or shallower discounting, whereas lower values indicate higher or steeper discounting. That is, higher values indicate little decrease in subjective value between the outcomes over the independent variable, whereas lower values indicate a large decrease in subjective value between the two outcomes over the independent variable. Because the smaller option always remained on the left for the slider questions, questions from Experiment 2 that involved losses were converted by subtracting the response from 100. This is account for the decrease in subjective value of the loss so that area differences between gains and losses could be directly compared.

To determine the relationship between flossing and discounting, statistical analyses were conducted using R 4.03 [21]. Because flossing questions were asked in a categorical manner rather than in a continuous one (i.e., how often you floss from these seven options rather than indicate how many days a year you floss), flossing frequency was treated as levels of a factor and a linear regression was conducted to determine how discounting changes as frequency of flossing decreases. That is, each group mean was compared to each other’s to determine differences between them.^1^ Daily flossing was used as the reference in the linear regression as it was expected that those who flossed daily would have the highest AUC_ord_ for delay discounting and lowest for probability discounting (i.e., least impulsivity/risk propensity), and thus would serve as a logical group to base comparisons from.

Post-hoc comparisons between all groups were also conducted using the emmeans package [22] with Holm-Bonferroni corrections to account for multiple comparisons. To analyze data from Experiment 1, AUC_ord_ was only calculated for the delays at 0.99 probability and probability at a delay of 1 day. This was to isolate the effects of delay relatively independent of probability and vice versa. To help ensure data quality, a slider question meant to determine attention of “Would you rather have $1000 immediately or $1 in a year?” was included in the survey. Values greater than 5% were excluded for purposes of analysis.

## Results

### Experiment 1

Demographic information is shown in Table 1. The sample (n = 584) was predominantly male (61.13%) and Caucasian (77.57%). The most commonly selected flossing frequency was flossing at least once a day (37.16%).

**Table 1:** Experiment 1 demographics and flossing

	*n*/mean	%/SD
Age	37.96	± 19.33
Income	51,468.44	± 41,623
Sex		
Female	222	38.01%
Male	357	61.13%
Other	5	0.86%
Ethnicity		
Asian	46	7.88%
Black/African	47	8.05%
Caucasian	453	77.57%
Hispanic/Latin	31	5.31%
Other	7	1.20%
Flossing frequency		
At least once a day	217	37.16%
At least once a week	185	31.68%
At least once a month	75	12.84%
At least once in 6 months	39	6.68%
At least once a year	21	3.60%
Less than once a year	47	8.05%

For discrete variables, the number of subjects and percentage of sample are included. For continuous variables, mean and SD are included

**Fig. 2:**
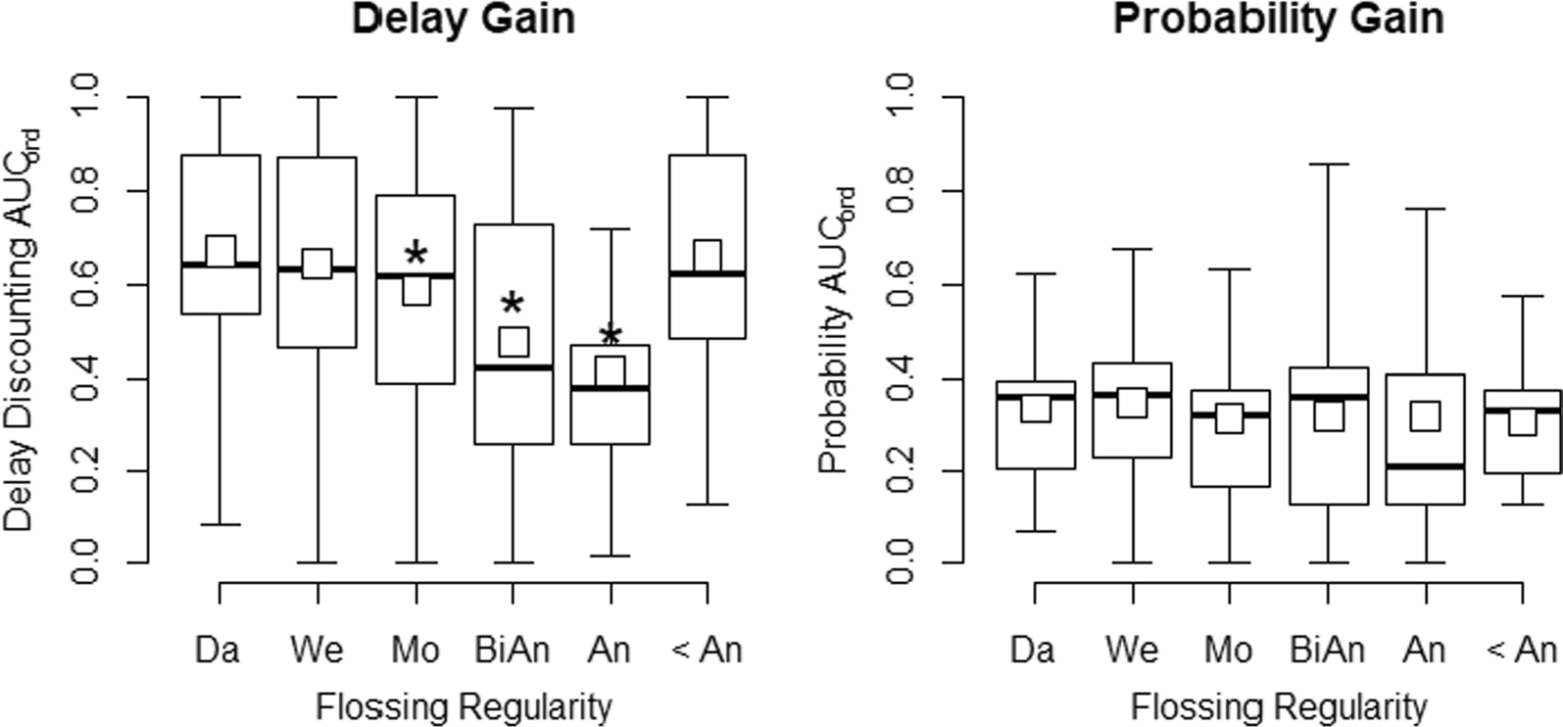
Boxplots of delay (left panel) and probability (right) discounting organized by frequency of flossing (x-axes) from Experiment 1. Discounting was calculated using area under the curve with ordinal scaling (AUC_ord_; see text for more details). Higher AUC_ord_ values for delay discounting indicate higher preference for the delayed outcome (i.e., lower impulsivity), whereas lower values of AUC_ord_ for probability discounting indicate higher preference for the certain option (i.e., lower riskiness). Squares represent the mean AUC_ord_, horizontal black lines represent the median, bottoms and tops of boxes represent the 25th and 75th percentiles respectively, and whiskers are 1.5 times interquartile range. Self-reported flossing frequency; Da: At least once a day. We: At least once a week. Mo: At least once a month. BiAn: At least once in 6 months. An: At least once a year. < An: Less than once a year. *Statistically significant decreases from mean AUC_ord_ of daily flossing at α = 0.05. Note that there were no significant increases from daily flossing

Discounting for delayed gains was related to self-reported flossing, *F*(5) = 7.502, *p* < 0.001. Regression outputs for both delay and probability discounting can be found in Table 2. Results of the regression of discounting of delayed gains indicated that there was a decrease in AUC_ord_ (i.e., higher preference for the smaller, sooner monetary gain) between daily flossing and less frequent flossing. That is, AUC_ord_ for delayed gains was higher for daily flossing relative to monthly, biannual, and yearly flossing. However, there was no significant difference between those who reported flossing daily, weekly, or less than once a year. By contrast, there was no relationship between flossing and probability discounting, *F*(5) = 0.891, *p* = 0.487. As shown in Table 1, group n tended to decrease as flossing frequency decreased. Figure 2 shows boxplots of delay and probability discounting as a function of self-reported flossing frequency. Post-hoc comparisons between all groups can be found in Table 3. Post-hoc comparisons generally agreed with the differences from daily flossing and other groups, although after corrections for multiple comparisons the difference between daily flossing and monthly flossing was no longer significant, *t*(578) = 2.46, *p* = 0.114.

**Table 2:** Experiment 1 regression of discounting on flossing

	Delayed gain			Probabilistic gain		
	Estimate	SE	*p*	Estimate	SE	*p*
(Intercept)	0.673***	0.018	< 0.001	0.335***	0.011	< 0.001
At least once a week	− 0.026	0.026	0.306	0.013	0.016	0.411
At least once a month	− 0.085*	0.035	0.014	− 0.021	0.028	0.333
At least once in 6 months	− 0.194***	0.045	< 0.001	− 0.017	0.028	0.538
At least once a year	− 0.259***	0.059	< 0.001	− 0.016	0.037	0.671
Less than once a year	− 0.012	0.042	0.773	− 0.028	0.026	0.279

Parameter estimates for delay/probability discounting of gains and frequency of flossing. The intercept is the mean of discounting at the group that responded as flossing at least daily. Daily flossing is the reference for other parameter estimates in so far that each estimate that is not the intercept represents a mean increase or decrease from the group that responded as flossing at least daily

*Estimate is significant from daily flossers at the 0.05 level

**Estimate is significant from daily flossers at the 0.01 level

***Estimate is significant from daily flossers at *p* < 0.001. SE: standard error. Note that the intercept being significant simply refers to AUC_ord_ for daily flossing being significantly different from zero

**Table 3:** Post-hoc comparisons between flossing frequencies and delay/probability discounting

Contrast	Delayed gains					Probabilistic gains				
	Estimate	SE	*df*	*t*	*p*	Estimate	SE	*df*	*t*	*p*
Da–We	0.027	0.026	578	1.024	1	− 0.013	0.016	578	− 0.823	1
Da–Mo	0.085	0.035	578	2.46	0.114	0.021	0.022	578	0.968	1
Da–BiAn	0.194*	0.045	578	4.327	< 0.001	0.017	0.028	578	0.617	1
Da–An	0.259*	0.059	578	4.386	< 0.001	0.016	0.037	578	0.425	1
Da–< An	0.012	0.042	578	0.288	1	0.028	0.026	578	1.083	1
We–Mo	0.059	0.035	578	1.658	0.587	0.034	0.022	578	1.549	1
We–BiAn	0.168*	0.046	578	3.69	0.003	0.031	0.029	578	1.076	1
We–An	0.233*	0.06	578	3.908	0.001	0.029	0.037	578	0.78	1
We–< An	− 0.015	0.042	578	− 0.343	1	0.042	0.027	578	1.571	1
Mo–BiAn	0.109	0.051	578	2.143	0.228	− 0.004	0.032	578	− 0.114	1
Mo–An	0.174	0.064	578	2.726	0.06	− 0.005	0.04	578	− 0.131	1
Mo–< An	− 0.073	0.048	578	− 1.522	0.643	0.007	0.03	578	0.24	1
BiAn–An	0.065	0.07	578	0.923	1	− 0.002	0.044	578	− 0.037	1
BiAn–< An	− 0.182*	0.056	578	− 3.26	0.012	0.011	0.035	578	0.31	1
An–< An	− 0.247*	0.068	578	− 3.642	0.003	0.012	0.043	578	0.294	1

Contrasts of all conditions based on the flossing/discounting models in Experiment 1. Multiple comparisons were corrected using the Holm–Bonferroni method. Self-reported flossing frequency; Da: At least once a day. We: At least once a week. Mo: At least once a month. BiAn: At least once in 6 months. An: At least once a year. < An: Less than once a year

*Statistically significant differences from mean AUC_ord_ by flossing frequency at α = .05. Contrast: groups being compared. Estimate: estimated difference between flossing frequencies. SE: standard error. *df*: degrees of freedom

### Experiment 2

Demographic information is shown in Table 4. As in Experiment 1, the sample (n = 321) was predominantly male (61.06%) and Caucasian (76.95%), and the most commonly selected flossing frequency was flossing at least once a day (33.96%). The proportions of subjects falling into each flossing frequency category were similar to those observed in Experiment 1.

**Table 4:** Experiment 2 demographics and flossing

	*n*/mean	%/SD
Age	37.96	± 19.33
Income	51,612.71	± 47,083
Sex		
Female	124	38.63%
Male	196	61.06%
Other	1	0.31%
Ethnicity		
Asian	21	6.54%
Black/African	27	8.41%
Caucasian	247	76.95%
Hispanic/Latin	23	7.17%
Other	3	0.93%
Flossing frequency		
At least once a day	109	33.96%
At least once a week	85	26.48%
At least once a month	38	11.84%
At least once in 6 months	35	10.90%
At least once a year	27	8.41%
Less than once a year	27	8.41%

For discrete variables, the number of subjects and percentage of sample are included. For continuous variables, mean and SD are included

Table 5 shows regression outputs of delay and probability discounting of gains and losses. Similar to Experiment 1, discounting of delayed gains was significant between self-reported flossing frequency, *F*(5) = 5.650, *p* < 0.001, but not for discounting of probabilistic gains, *F*(5) = 1.413, *p* = 0.219. For delay discounting, significant differences were observed for the same comparisons as in Experiment 1. Also similar to Experiment 1, probability discounting did not appear to be related to reported flossing frequency. Post-hoc comparisons were congruent with the regression analysis between daily flossing and other flossing frequencies. All post-hoc comparisons between flossing frequencies and discounting type can be found in Table 6.

**Table 5:** Experiment 2 regression of discounting on flossing

	Delayed gain			Probabilistic gain		
	Estimate	SE	*p*	Estimate	SE	*p*
(Intercept)	0.692***	0.029	< 0.001	0.354***	0.018	< 0.001
At least once a week	− 0.042	0.044	0.339	− 0.000	0.027	0.994
At least once a month	− 0.218***	0.057	< 0.001	− 0.056	0.035	0.114
At least once in 6 months	− 0.219***	0.059	< 0.001	− 0.045	0.036	0.220
At least once a year	− 0.194**	0.064	0.003	− 0.058	0.040	0.147
Less than once a year	− 0.074	0.065	0.260	0.032	0.040	0.426

	Delayed loss			Probabilistic loss		
(Intercept)	0.814***	0.030	< 0.001	0.536***	0.020	< 0.001
At least once a week	− 0.068	0.045	0.128	0.024	0.030	0.427
At least once a month	− 0.192*	0.059	0.012	− 0.058	0.039	0.135
At least once in 6 months	− 0.202***	0.060	< 0.001	− 0.062	0.040	0.125
At least once a year	− 0.085	0.066	0.197	0.059	0.044	0.176
Less than once a year	− 0.100	0.067	0.135	0.074	0.044	0.097

Parameter estimates for delay/probability discounting of gains and frequency of flossing. Discounting of gains are in top half of the table, discounting of losses is in the bottom path of the table. The intercept is the mean of discounting at the group that responded as flossing at least daily. Daily flossing is the reference for other parameter estimates in so far that each estimate that is not the intercept represents a mean increase or decrease from the group that responded as flossing at least daily

*Estimate is significant from daily flossers at the 0.05 level

**Estimate is significant from daily flossers at the 0.01 level

***Estimate is significant from daily flossers at *p* < 0.001. SE: Standard error. Note that the intercept being significant simply refers to AUC_ord_ for daily flossing being significantly different from zero

**Table 6:** Post-hoc comparisons between flossing frequencies and delay/probability discounting

Contrast	Delayed gain					Probabilistic gain				
	Estimate	SE	*df*	*t*	*p*	Estimate	SE	*df*	*t*	*p*
Da–We	0.042	0.044	318	0.958	1	0	0.027	318	0.008	1
Da–Mo	0.218*	0.057	318	3.819	0.002	0.056	0.035	318	1.583	1
Da–BiAn	0.219*	0.059	318	3.729	0.003	0.045	0.036	318	1.228	1
Da–An	0.194*	0.064	318	3.031	0.034	0.058	0.04	318	1.455	1
Da–< An	0.074	0.065	318	1.13	1	− 0.032	0.04	318	− 0.798	1
We–Mo	0.176*	0.059	318	2.981	0.037	0.055	0.036	318	1.523	1
We–BiAn	0.177*	0.061	318	2.921	0.041	0.044	0.037	318	1.184	1
We–An	0.153	0.066	318	2.315	0.212	0.057	0.041	318	1.41	1
We–< An	0.032	0.067	318	0.475	1	− 0.032	0.041	318	− 0.782	1
Mo–BiAn	0.002	0.071	318	0.022	1	− 0.011	0.044	318	− 0.254	1
Mo–An	− 0.023	0.076	318	− 0.309	1	0.002	0.047	318	0.041	1
Mo–< An	− 0.144	0.076	318	− 1.891	0.536	− 0.088	0.047	318	− 1.864	0.949
BiAn–An	− 0.025	0.077	318	− 0.323	1	0.013	0.047	318	0.274	1
BiAn–< An	− 0.146	0.078	318	− 1.878	0.536	− 0.077	0.048	318	− 1.6	1
An–< An	− 0.121	0.082	318	− 1.48	0.98	− 0.089	0.05	318	− 1.777	1

	Delayed loss					Probabilistic loss				
Da–We	0.068	0.045	318	1.525	1	− 0.024	0.03	318	− 0.796	1
Da–Mo	0.192*	0.059	318	3.278	0.016	0.058	0.039	318	1.499	1
Da–BiAn	0.202*	0.061	318	3.346	0.014	0.062	0.04	318	1.536	1
Da–An	0.085	0.066	318	1.293	1	− 0.059	0.044	318	− 1.356	1
Da–< An	0.1	0.067	318	1.499	1	− 0.074	0.044	318	− 1.666	0.871
We–Mo	0.124	0.061	318	2.04	0.507	0.082	0.04	318	2.036	0.444
We–BiAn	0.134	0.063	318	2.144	0.427	0.085	0.042	318	2.059	0.444
We–An	0.017	0.068	318	0.249	1	− 0.036	0.045	318	− 0.792	1
We–< An	0.032	0.069	318	0.464	1	− 0.05	0.046	318	− 1.103	1
Mo–BiAn	0.01	0.073	318	0.139	1	0.003	0.049	318	0.069	1
Mo–An	− 0.107	0.078	318	− 1.378	1	− 0.118	0.052	318	− 2.284	0.282
Mo–< An	− 0.092	0.079	318	− 1.172	1	− 0.132	0.052	318	− 2.542	0.164
BiAn–An	− 0.117	0.079	318	− 1.481	1	− 0.121	0.053	318	− 2.308	0.282
BiAn–< An	− 0.102	0.08	318	− 1.279	1	− 0.136	0.053	318	− 2.561	0.164
An–< An	0.015	0.084	318	0.178	1	− 0.015	0.056	318	− 0.263	1

Contrasts of all conditions based on the flossing/discounting models in Experiment 2. Multiple comparisons were corrected using the Holm–Bonferroni method. Discounting of gains is on the top half of the table, whereas discounting of losses in on the bottom half of the table. Self-reported flossing frequency; Da: At least once a day. We: At least once a week. Mo: At least once a month. BiAn: At least once in 6 months. An: At least once a year. < An: Less than once a year

*Statistically significant differences from mean AUC_ord_ by flossing frequency at α = 0.05. Contrast: groups being compared. Estimate: estimated difference between flossing frequencies. SE: standard error. *df*: degrees of freedom

**Fig. 3:**
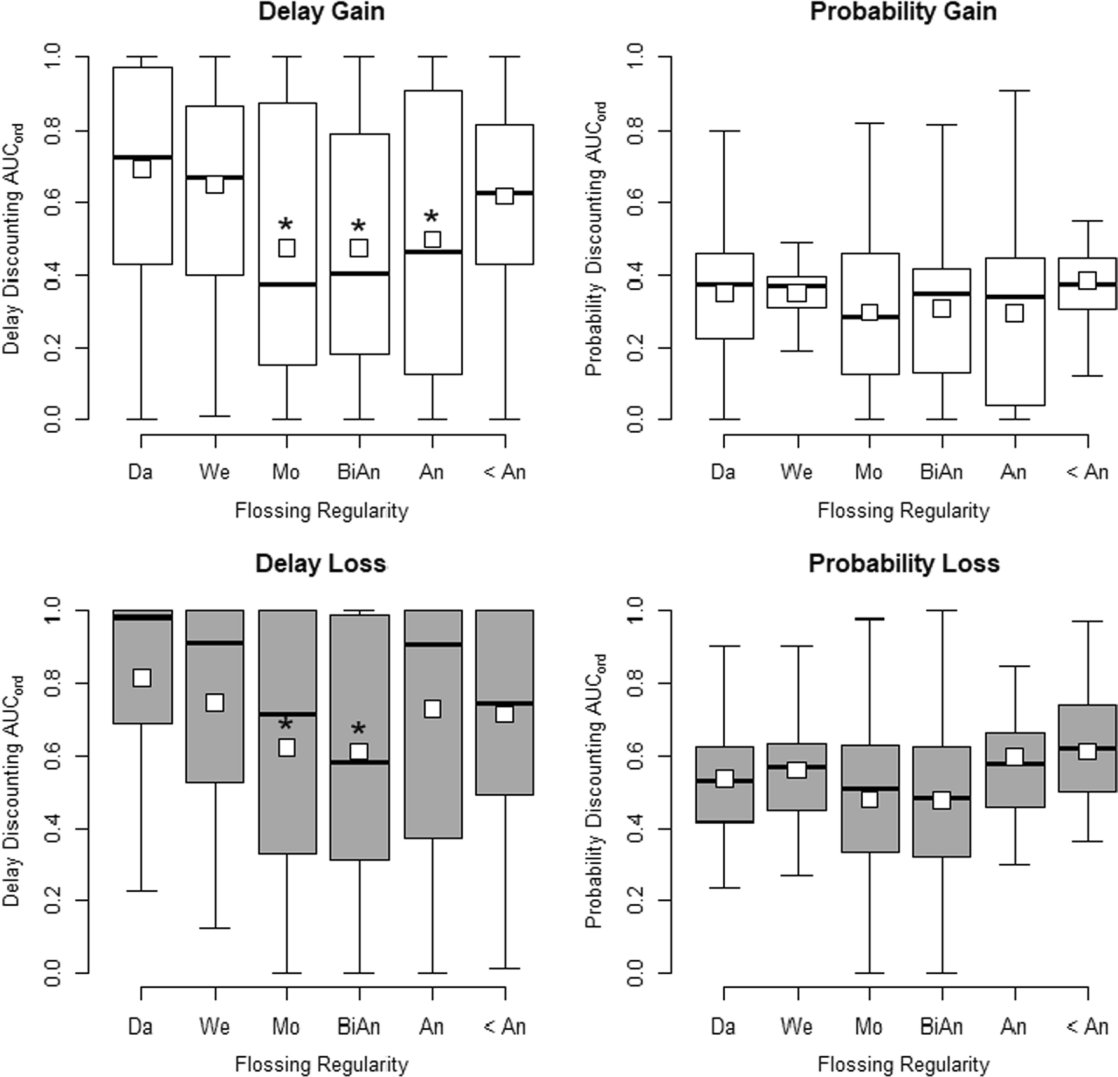
Boxplots of delay (left panels) and probability (right panels) discounting organized by frequency of flossing (x-axes) from Experiment 2. Top panels/white boxes indicate discounting for monetary gains, bottom panels/grey boxes indicate discounting for monetary losses. Discounting was calculated using area under the curve with ordinal scaling (AUC_ord_; see text for more details). Higher AUC_ord_ values for delay discounting indicate higher preference for the delayed outcome (i.e., lower impulsivity), whereas lower values of AUC_ord_ for probability discounting indicate higher preference for the certain option (i.e., lower riskiness). Squares represent the mean AUC_ord_, horizontal black lines represent the median, bottoms and tops of boxes represent the 25th and 75th percentiles respectively, and whiskers are 1.5 times interquartile range. Self-reported flossing frequency; Da: At least once a day. We: At least once a week. Mo: At least once a month. BiAn: At least once in 6 months. An: At least once a year. < An: Less than once a year. *Statistically significant decreases from mean AUC_ord_ of daily flossing at α = 0.05. Note that there were no significant increases from daily flossing

There was a significant relationship for discounting of delayed, *F*(5) = 3.561, *p* = 0.004, and probabilistic, *F*(5) = 2.580, *p* = 0.026, losses by flossing frequency. Nevertheless, there were no significant differences related to daily flossing and other flossing frequencies based on AUC_ord_ of probabilistic losses determined by the regression output, nor were there any significant post-hoc differences between flossing frequency and discounting of probabilistic losses after correction for multiple comparisons. Discounting of delayed losses was similar to delayed gains, with the exception that of annual flossing was not significantly different from daily flossing. Boxplots of delay and probability discounting for gains and losses by flossing frequency can be found in Fig. 3. Reported flossing also followed the same pattern as Experiment 1, with the sizes of groups decreasing as frequency of flossing decreased.

## Discussion

Experiments 1 and 2 show that people who engage in daily interdental cleaning discount delayed gains less than people who engage in interdental cleaning occasionally throughout a year. This finding is consistent with prior studies of the relationship between delay discounting and other preventive health behaviors, including attending primary care visits [14, 23], medication adherence [24], and social distancing during the COVID-19 pandemic [25]. It is also consistent with the preponderance of work in delay discounting and health behavior, which shows that greater discounting of delayed gains is associated with behavioral health problems such as substance use disorders and gambling [26].

In contrast, the present studies did not find support for a relationship between probabilistic discounting and frequency of interdental cleaning. This too, is consistent with weaker support for a relationship between probability discounting and cocaine use disorder [27], smoking [28], and obesity [29, 30]. Taken together, these results suggest that probability discounting is less robust as a predictor of health behavior. Further, the concordance in results across qualitatively different health behaviors suggests common processes underly human decision-making that affects health [12, 13].

One implication of the present finding involves interventions that were developed to improve health behavior by affecting discounting processes. Such interventions may be useful for improving interdental cleaning regularity, even if they were originally developed to address a different behavioral health problem. One especially promising intervention of this kind is episodic future thinking, in which a person imagines future autobiographical events [31]. Laboratory studies have shown that episodic future thinking can decrease discounting of delayed rewards [32, 33]. Although the evidence demonstrating that episodic future thinking can produce lasting change in health behavior is limited, small early studies show promising results in the domains of medication adherence [24] and smoking cessation [34]. An intervention in which patients are simply coached to practice vividly imagining a future in which they have good oral health because they flossed is seemingly practical. As such, it appears worthy of systematic evaluation. Another candidate intervention is exposure to nature. Viewing images of nature prior to completing a delay discounting task reduces discounting [35]. However, translational work in this nascent area is especially limited (see Berry et al. [36] for a brief review), and the manner in which this could be implemented in practice to increase the likelihood that people will include interdental cleaning in their oral health routine is not clear.

The present results also suggest that interdental cleaning could serve as an important target behavior for evaluating concepts and procedures that relate to health decisions and behaviors. It may seem that the population rate of flossing is low (32%; [3]). However, it is important to view this rate in light of the current and historical methods used to promote interdental cleaning. As described in the introduction, regular interdental cleaning is largely a product of recommendations, training, and rules provided by trusted dental professionals and close relatives. Any intervention that is developed for promoting interdental cleaning that better harness the available social supports should be considered a worthy candidate for generalization to other health behaviors. Further, fact that 32% of people floss regularly based on recommendations and rules could be viewed as a good outcome. More research on characteristics and practices of individuals who engage in interdental cleaning on a daily basis are worthy of investigation and also potentially applicable to other health behaviors. In this way, studies of interdental cleaning could be a source of novel and generalizable ideas that relate to the promotion of a wide variety of preventive health behaviors.

This study has several limitations, notably that it used crowd-sourced samples and that interdental cleaning regularity could not be verified. However, the similarity between the subject reports in the present study and those of Fleming et al. [3] adds confidence in our measure of interdental cleaning. Other potential problems with crowd-sourced samples were mitigated by other elements of the study design. This includes the initial comprehension check to be able to access the study, and the embedded attention check used to screen out bots or potentially inattentive responders (i.e., those trying to complete the survey as quickly as possible). In the present study, probability discounting, delay discounting, and self-reported interdental cleaning regularity were included in a battery of other experimental and demographic questions, which obscures potential influence by experimenter-hypothesized relationships. Confidence in the results is improved by the replication across two experiments with independent samples using different, but similar, discounting tasks. Nevertheless, independent replication of the relationship between discounting and interdental cleaning regularity is an important step for future research in this domain. Finally, the present study is silent with respect to causality between flossing and discounting, as well as whether other measures of impulsivity would show a similar relationship (e.g., [37]).

## Conclusion

The present study expands the range of health behaviors relevant to discounting to include interdental cleaning. This expansion has relevance to the development of interventions designed to promote interdental cleaning, and suggests that preventive oral health practices offer a valuable context in which to generate and evaluate behavioral health interventions.

## Supplementary Information

**Additional file 1:** Experimental instructions and question formats for Experiment 1.

**Additional file 2:** Experimental instructions and question formats for Experiment 2.

## Data Availability

Raw data are available for public access via openICPSR. Data used in the analyses for Experiment 1 are available at https://doi.org/10.3886/E162321V1. Data used in the analyses for Experiment 2 are available at https://doi.org/10.3886/E162322V1 (Rzeszutek & DeFulio, 2022b).

## Abbreviation

AUC_ord_: Area under the curve with ordinal scaling

## Footnotes

1 The *F* statistic from this type of linear regression (i.e., comparing categorical variables) is equivalent to the *F* statistic from an ANOVA.
