# Supplementary material for: Delay discounting, probability discounting, and interdental cleaning frequency
Source: BMC Oral Health. 2022 Jul 29;22:315. doi: 10.1186/s12903-022-02328-6 (PMC9335449; doi:10.1186/s12903-022-02328-6)
Supplement: Supplementary file 1 — Additional file 1: Experimental instructions and question formats for Experiment 1. [file 12903_2022_2328_MOESM1_ESM.pdf]

Preamble

Prior to beginning the study, you will be required to complete 6 slider/percentage training questions, followed by a 4-question percentage comprehension test. If you do not pass the percentage comprehension test, you will be excluded from the study and will only be compensated $0.25 following a captcha. Please pay careful attention to the following examples and questions. This section should take less than 5 minutes. If you exit out of this survey, you will not be able to reopen it.

Slider Use Example

Prior to beginning the experiment, you will be asked a series of questions involving ratios and the corresponding percentages associated with them. You will be using a slider bar to identify your answer. Understanding percentages is key to this study, as most questions will be in this format. Every answer will require to move or click the slider, even if it is already at the value you wish to choose. To continue to the task, please move the slider off of 50%, and then back to 50% as demonstrated below. To use the slider, click the circle in the center of the bar, then drag it to the desired location(s).

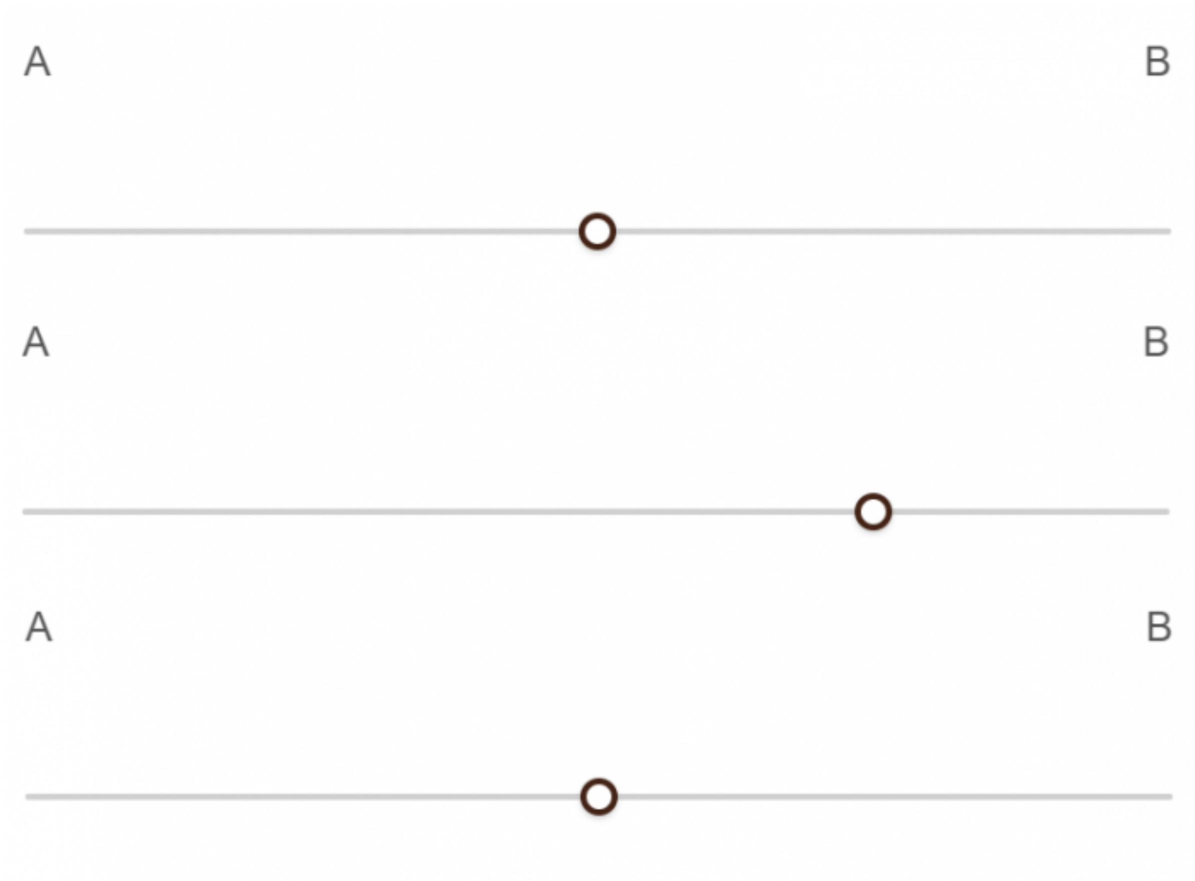

Please move the slider off of **<u>50%</u>** and back on to **<u>50%</u>** now

A B

0 25 50 75 100

Teaching A over B

If you were to choose A 100% of the time and B 0% of the time, you would move the slider to the leftmost position seen below.

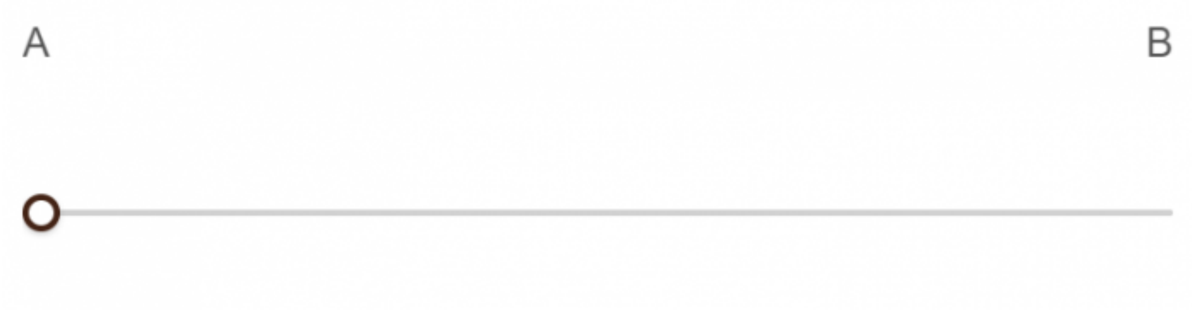

Please move the slider as if though you were choosing **A 100%** of the time and **B 0%** of the time

A				B
0	25	50	75	100

Teaching B over A

If you were to choose B 100% of the time and A 0% of the time, you would move the slider to the rightmost position seen below.

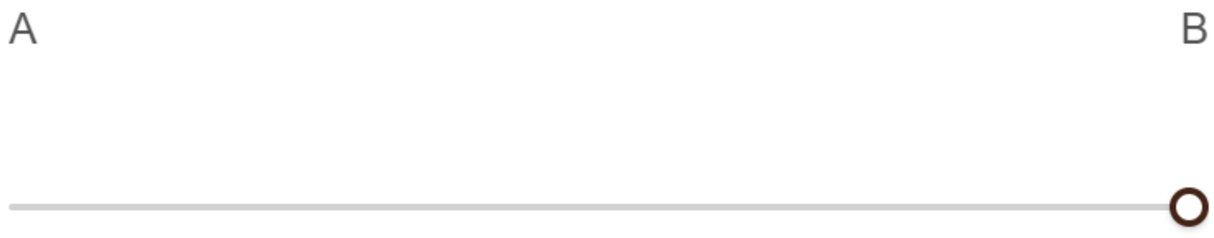

Please move the slider as if though you were choosing **B 100%** of the time and **A 0%** of the time

A B

0 25 50 75 100

Teaching 75% A

If you were to choose A 75% of the time and B 25% of the time, you would move the slider to the position seen below.

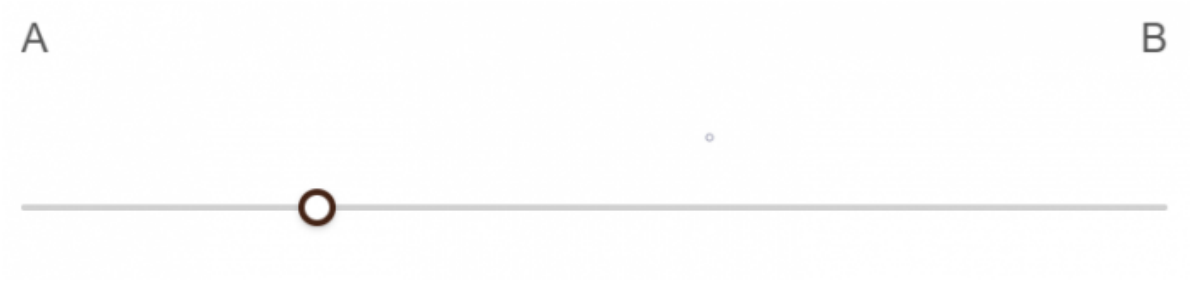

Please move the slider as if though you were choosing **A 75%** of the time and **B 25%** of the time

A B

0 25 50 75 100

Teaching 50/50

If you were to choose A 50% of the time and B 50% of the time, you would move the slider to the position seen below.

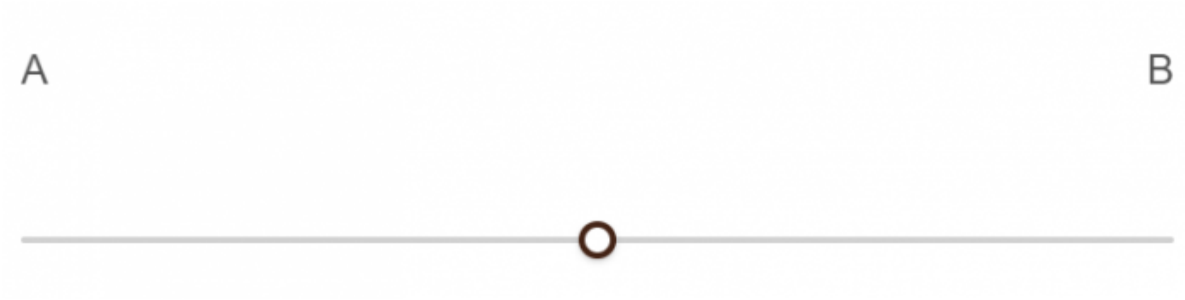

Please move the slider as if though you were choosing **A 50%** of the time and **B 50%** of the time

A B

0 25 50 75 100

Teaching 75% B

If you were to choose B 75% of the time and A 25% of the time, you would move the slider to the position seen below.

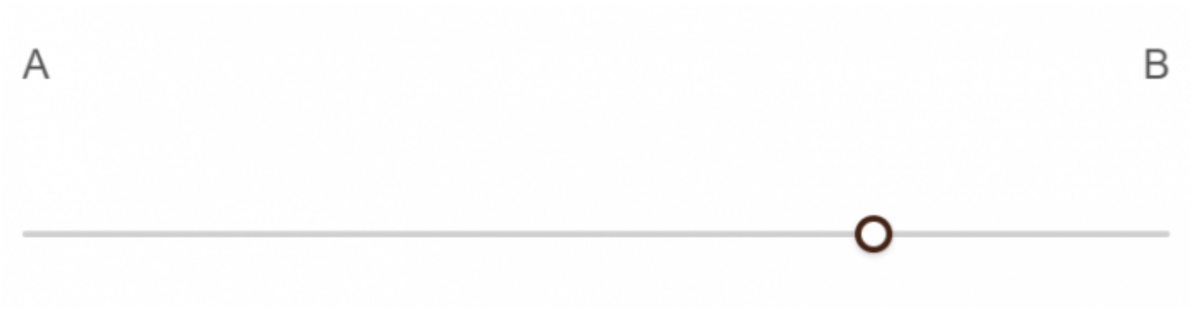

Please move the slider as if though you were choosing **B 75%** of the time and **A 25%** of the time

A				B
0	25	50	75	100

Slider Test Break

You will now be tested on the previous content. Understanding percentages and the slider is necessary to continue on to the study. If you do not get all the four following questions correct you will be considered ineligible and removed from the study. You will only have one chance to answer these correctly, so please pay attention to the questions carefully. You will not be eligible for the $3.00 bonus if you are removed from the study in this way.

Testing Block

If you would **100%** choose **A** over B, move the slider to the correct spot

A				B
0	25	50	75	100

If you would choose **A** **75%** of the time and **B** **25%** of the time, move the slider to the correct spot

A				B
0	25	50	75	100

If you would choose **B** **75%** of the time and **A** **25%** of the time, move the slider to the correct spot

A				B
0	25	50	75	100

If you would **100%** choose **B** over A, move the slider to the correct spot

A				B
0	25	50	75	100

completion break fail

You did not successfully complete the screener. The next page has your completion ID, please follow the instructions on that page to receive the completion ID and complete the survey. Prior to this, please complete the Captcha.

I'm not a robot

reCAPTCHA
Privacy - Terms

Completion ID Fail

You did not pass the screener question.

Your completion ID is ${e://Field/Fail%20ID}, make sure you save this number as this indicates you have completed the survey.

Please paste this number ${e://Field/Fail%20ID} into MTurk before pressing next . If you press next without saving the number, you may not be compensated for your participation.

Because you will not be moving onto the rest of the survey, you will only be compensated $0.25.

When you press next, you will have completed the survey.

Discounting Gain Questions Break

You have successfully completed the screener.

You will now be asked a series of hypothetical questions regarding monetary choices. Please read the questions carefully and respond as if the outcomes are real.

Monetary 1-day LL

What is the likelihood that you would select a **99%** chance of gaining **$500** in **1 day** over a **99%** chance of gaining **$1,000** in **1 day**?

99% chance of gaining $500 in 1 day		99% chance of gaining $1,000 in 1 day
	○	

What is the likelihood that you would select a **99%** chance of gaining **$500** in **1 day** over a **80%** chance of gaining **$1,000** in **1 day**?

99% chance of gaining $500 in 1 day	80% chance of gaining $1,000 in 1 day

What is the likelihood that you would select a **99%** chance of gaining **$500** in **1 day** over a **50%** chance of gaining **$1,000** in **1 day**?

99% chance of gaining $500 in 1 day	50% chance of gaining $1,000 in 1 day

What is the likelihood that you would select a **99%** chance of gaining **$500** in **1 day** over a **20%** chance of gaining **$1,000** in **1 day**?

99% chance of gaining $500 in 1 day	20% chance of gaining $1,000 in 1 day

What is the likelihood that you would select a **99%** chance of gaining **$500** in **1 day** over a **1%** chance of gaining **$1,000** in **1 day**?

99% chance of gaining $500 in 1 day		1% chance of gaining $1,000 in 1 day
	○	

Monetary 1 month LL

What is the likelihood that you would select a **99%** chance of gaining **$500** in **1 day** over a **99%** chance of gaining **$1,000** in **1 month**?

99% chance of gaining $500 in 1 day		99% chance of gaining $1,000 in 1 month
	○	

What is the likelihood that you would select a **99%** chance of gaining **$500** in **1 day** over a **80%** chance of gaining **$1,000** in **1 month**?

99% chance of gaining $500 in 1 day		80% chance of gaining $1,000 in 1 month
	○	

What is the likelihood that you would select a **99%** chance of gaining **$500** in **1 day** over a **50%** chance of gaining **$1,000** in **1 month**?

99% chance of gaining $500 in 1 day	50% chance of gaining $1,000 in 1 month

What is the likelihood that you would select a **99%** chance of gaining **$500** in **1 day** over a **20%** chance of gaining **$1,000** in **1 month**?

99% chance of gaining $500 in 1 day	20% chance of gaining $1,000 in 1 month

What is the likelihood that you would select a **99%** chance of gaining **$500** in **1 day** over a **1%** chance of gaining **$1,000** in **1 month**?

99% chance of gaining $500 in 1 day	1% chance of gaining $1,000 in 1 month

Monetary 6 months LL

What is the likelihood that you would select a **99%** chance of gaining **$500** in **1 day** over a **99%** chance of gaining **$1,000** in **6 months**?

99% chance of gaining $500 in 1 day	99% chance of gaining $1,000 in 6 months

What is the likelihood that you would select a **99%** chance of gaining **$500** in **1 day** over a **80%** chance of gaining **$1,000** in **6 months**?

99% chance of gaining $500 in 1 day	80% chance of gaining $1,000 in 6 months

What is the likelihood that you would select a **99%** chance of gaining **$500** in **1 day** over a **50%** chance of gaining **$1,000** in **6 months**?

99% chance of gaining $500 in 1 day	50% chance of gaining $1,000 in 6 months

What is the likelihood that you would select a **99%** chance of gaining **$500** in **1 day** over a **20%** chance of gaining **$1,000** in **6 months**?

99% chance of gaining $500 in 1 day	20% chance of gaining $1,000 in 6 months

What is the likelihood that you would select a **99%** chance of gaining **$500** in **1 day** over a **1%** chance of gaining **$1,000** in **6 months**?

99% chance of gaining $500 in 1 day	1% chance of gaining $1,000 in 6 months

Monetary 2 years LL

What is the likelihood that you would select a **99%** chance of gaining **$500** in **1 day** over a **99%** chance of gaining **$1,000** in **2 years**?

99% chance of gaining $500 in 1 day	99% chance of gaining $1,000 in 2 years

What is the likelihood that you would select a **99%** chance of gaining **$500** in **1 day** over a **80%** chance of gaining **$1,000** in **2 years**?

99% chance of gaining $500 in 1 day	80% chance of gaining $1,000 in 2 years

What is the likelihood that you would select a **99%** chance of gaining **$500** in **1 day** over a **50%** chance of gaining **$1,000** in **2 years**?

99% chance of gaining $500 in 1 day	50% chance of gaining $1,000 in 2 years

What is the likelihood that you would select a **99%** chance of gaining **$500** in **1 day** over a **20%** chance of gaining **$1,000** in **2 years**?

99% chance of gaining $500 in 1 day	20% chance of gaining $1,000 in 2 years

What is the likelihood that you would select a **99%** chance of gaining **$500** in **1 day** over a **1%** chance of gaining **$1,000** in **2 years** ?

99% chance of gaining $500 in 1 day	1% chance of gaining $1,000 in 2 years

Monetary 5 years LL

What is the likelihood that you would select a **99%** chance of gaining **$500** in **1 day** over a **99%** chance of gaining **$1,000** in **5 years**?

99% chance of gaining $500 in 1 day	99% chance of gaining $1,000 in 5 years

What is the likelihood that you would select a **99%** chance of gaining **$500** in **1 day** over a **80%** chance of gaining **$1,000** in **5 years**?

99% chance of gaining $500 in 1 day	80% chance of gaining $1,000 in 5 years

What is the likelihood that you would select a **99%** chance of gaining **$500** in **1 day** over a **50%** chance of gaining **$1,000** in **5 years**?

99% chance of gaining $500 in 1 day	50% chance of gaining $1,000 in 5 years

What is the likelihood that you would select a **99%** chance of gaining **$500** in **1 day** over a **20%** chance of gaining **$1,000** in **5 years**?

99% chance of gaining $500 in 1 day	20% chance of gaining $1,000 in 5 years

What is the likelihood that you would select a **99%** chance of gaining **$500** in **1 day** over a **1%** chance of gaining **$1,000** in **5 years**?

99% chance of gaining $500 in 1 day	1% chance of gaining $1,000 in 5 years

Virus Treatment Free

Imagine there is a viral outbreak of a new virus that can manifest as any number of five moderate symptoms: headache, nausea, cough,

fever, diarrhea. Also imagine that the severity of each these symptoms is relatively moderate to you, such that what you consider a moderate headache is equivalent to moderate nausea, moderate cough, and so on. Typically, symptoms of the virus will worsen over time, but imagine you have experienced the symptoms in the following questions for two days. Increased symptom severity and potential death from the virus typically occurs two weeks after the first symptoms appear, although medical care can decrease the risk of death. Answer the following questions as if the situation were real. Note that there is no wrong way to answer these questions.

1 of 5 symptoms free

You've displayed **1 of 5 symptoms** of a new virus, with a **1% fatality rate**. How likely are you to see a healthcare professional?

0% chance of seeking healthcare	1 of 5 symptoms, 1% fatality rate	100% chance of seeking healthcare
	○	

You've displayed **1 of 5 symptoms** of a new virus, with a **3% fatality rate**. How likely are you to see a healthcare professional?

0% chance of seeking healthcare	1 of 5 symptoms, 3% fatality rate	100% chance of seeking healthcare

You've displayed **1 of 5 symptoms** of a new virus, with a **9% fatality rate**. How likely are you to see a healthcare professional?

0% chance of seeking healthcare	1 of 5 symptoms, 9% fatality rate	100% chance of seeking healthcare

You've displayed **1 of 5 symptoms** of a new virus, with a **18% fatality rate**. How likely are you to see a healthcare professional?

0% chance of seeking healthcare	1 of 5 symptoms, 18% fatality rate	100% chance of seeking healthcare

You've displayed **1 of 5 symptoms** of a new virus, with a **36% fatality rate**. How likely are you to see a healthcare professional?

0% chance of seeking healthcare	1 of 5 symptoms, 36% fatality rate	100% chance of seeking healthcare
	○	

3 of 5 symptoms free

You've displayed **3 of 5 symptoms** of a new virus, with a **1% fatality rate**. How likely are you to see a healthcare professional?

0% chance of seeking healthcare	3 of 5 symptoms, 1% fatality rate	100% chance of seeking healthcare
	○	

You've displayed **3 of 5 symptoms** of a new virus, with a **3% fatality rate**. How likely are you to see a healthcare professional?

0% chance of seeking healthcare	3 of 5 symptoms, 3% fatality rate	100% chance of seeking healthcare

You've displayed **3 of 5 symptoms** of a new virus, with a **9% fatality rate**. How likely are you to see a healthcare professional?

0% chance of seeking healthcare	3 of 5 symptoms, 9% fatality rate	100% chance of seeking healthcare

You've displayed **3 of 5 symptoms** of a new virus, with a **18% fatality rate**. How likely are you to see a healthcare professional?

0% chance of seeking healthcare	3 of 5 symptoms, 18% fatality rate	100% chance of seeking healthcare

You've displayed **3 of 5 symptoms** of a new virus, with a **36% fatality rate**. How likely are you to see a healthcare professional?

0% chance of seeking healthcare	3 of 5 symptoms, 36% fatality rate	100% chance of seeking healthcare
	○	

5 of 5 symptoms free

You've displayed **5 of 5 symptoms** of a new virus, with a **1% fatality rate**. How likely are you to see a healthcare professional?

0% chance of seeking healthcare	5 of 5 symptoms, 1% fatality rate	100% chance of seeking healthcare
	○	

You've displayed **5 of 5 symptoms** of a new virus, with a **3% fatality rate**. How likely are you to see a healthcare professional?

0% chance of seeking healthcare	5 of 5 symptoms, 3% fatality rate	100% chance of seeking healthcare
	○	

You've displayed **5 of 5 symptoms** of a new virus, with a **9% fatality rate**. How likely are you to see a healthcare professional?

0% chance of seeking healthcare	5 of 5 symptoms, 9% fatality rate	100% chance of seeking healthcare
	○	

You've displayed **5 of 5 symptoms** of a new virus, with a **18% fatality rate**. How likely are you to see a healthcare professional?

0% chance of seeking healthcare	5 of 5 symptoms, 18% fatality rate	100% chance of seeking healthcare
	○	

You've displayed **5 of 5 symptoms** of a new virus, with a **36% fatality rate**. How likely are you to see a healthcare professional?

0% chance of seeking healthcare	5 of 5 symptoms, 36% fatality rate	100% chance of seeking healthcare

Virus Treatment Cost

Imagine there is a viral outbreak of a new virus that can manifest as any number of five moderate symptoms: headache, nausea, cough, fever, diarrhea. Also imagine that the severity of each these symptoms is relatively moderate to you, such that what you consider a moderate headache is equivalent to moderate nausea, moderate cough, and so on. Typically, symptoms of the virus will worsen over time, but imagine you have experienced the symptoms in the following questions for two days. Increased symptom severity and potential death from the virus typically occurs two weeks after the first symptoms appear, although medical care can decrease the risk of death. However, there is a cost associated with treatment for the virus.

Answer the following questions as if the situation were real. Note that there is no wrong way to answer these questions.

1 of 5 symptoms 5000

You've displayed **1 of 5 symptoms** of a new virus, with a **1% fatality rate**. **Treatment for the virus will cost you a total of $5,000**. How likely are you to see a healthcare professional?

0% chance of seeking healthcare at $5,000 treatment	1 of 5 symptoms, 1% fatality rate	100% chance of seeking healthcare at $5,000 treatment

You've displayed **1 of 5 symptoms** of a new virus, with a **3% fatality rate**. **Treatment for the virus will cost you a total of $5,000**. How likely are you to see a healthcare professional?

0% chance of seeking healthcare at $5,000 treatment	1 of 5 symptoms, 3% fatality rate	100% chance of seeking healthcare at $5,000 treatment

You've displayed **1 of 5 symptoms** of a new virus, with a **9% fatality rate**. **Treatment for the virus will cost you a total of $5,000**. How likely are you to see a healthcare professional?

0% chance of seeking healthcare at $5,000 treatment	1 of 5 symptoms, 9% fatality rate	100% chance of seeking healthcare at $5,000 treatment

You've displayed **1 of 5 symptoms** of a new virus, with a **18% fatality rate**. **Treatment for the virus will cost you a total of $5,000**. How likely are you to see a healthcare professional?

0% chance of seeking healthcare at $5,000 treatment	1 of 5 symptoms, 18% fatality rate	100% chance of seeking healthcare at $5,000 treatment

You've displayed **1 of 5 symptoms** of a new virus, with a **36% fatality rate**. **Treatment for the virus will cost you a total of $5,000**. How likely are you to see a healthcare professional?

0% chance of seeking healthcare at $5,000 treatment	1 of 5 symptoms, 36% fatality rate	100% chance of seeking healthcare at $5,000 treatment

3 of 5 symptoms 5000

You've displayed **3 of 5 symptoms** of a new virus, with a **1% fatality rate**. **Treatment for the virus will cost you a total of $5,000**. How likely are you to see a healthcare professional?

0% chance of seeking healthcare at $5,000 treatment	3 of 5 symptoms, 1% fatality rate	100% chance of seeking healthcare at $5,000 treatment

You've displayed **3 of 5 symptoms** of a new virus, with a **3% fatality rate**. **Treatment for the virus will cost you a total of $5,000**. How likely are you to see a healthcare professional?

0% chance of seeking healthcare at $5,000 treatment	3 of 5 symptoms, 3% fatality rate	100% chance of seeking healthcare at $5,000 treatment
	○	

You've displayed **3 of 5 symptoms** of a new virus, with a **9% fatality rate**. **Treatment for the virus will cost you a total of $5,000**. How likely are you to see a healthcare professional?

0% chance of seeking healthcare at $5,000 treatment	3 of 5 symptoms, 9% fatality rate	100% chance of seeking healthcare at $5,000 treatment
	○	

You've displayed **3 of 5 symptoms** of a new virus, with a **18% fatality rate**. **Treatment for the virus will cost you a total of $5,000**. How likely are you to see a healthcare professional?

0% chance of seeking healthcare at $5,000 treatment	3 of 5 symptoms, 18% fatality rate	100% chance of seeking healthcare at $5,000 treatment

You've displayed **3 of 5 symptoms** of a new virus, with a **36% fatality rate**. **Treatment for the virus will cost you a total of $5,000**. How likely are you to see a healthcare professional?

0% chance of seeking healthcare at $5,000 treatment	3 of 5 symptoms, 36% fatality rate	100% chance of seeking healthcare at $5,000 treatment

5 of 5 symptoms 5000

You've displayed **5 of 5 symptoms** of a new virus, with a **1% fatality rate**. **Treatment for the virus will cost you a total of $5,000**. How likely are you to see a healthcare professional?

0% chance of seeking healthcare at $5,000 treatment	5 of 5 symptoms, 1% fatality rate	100% chance of seeking healthcare at $5,000 treatment

You've displayed **5 of 5 symptoms** of a new virus, with a **3% fatality rate**. **Treatment for the virus will cost you a total of $5,000**. How likely are you to see a healthcare professional?

0% chance of seeking healthcare at $5,000 treatment	5 of 5 symptoms, 3% fatality rate	100% chance of seeking healthcare at $5,000 treatment

You've displayed **5 of 5 symptoms** of a new virus, with a **9% fatality rate**. **Treatment for the virus will cost you a total of $5,000**. How likely are you to see a healthcare professional?

0% chance of seeking healthcare at $5,000 treatment	5 of 5 symptoms, 9% fatality rate	100% chance of seeking healthcare at $5,000 treatment

You've displayed **5 of 5 symptoms** of a new virus, with a **18% fatality rate**. **Treatment for the virus will cost you a total of $5,000**. How likely are you to see a healthcare professional?

0% chance of seeking healthcare at $5,000 treatment	5 of 5 symptoms, 18% fatality rate	100% chance of seeking healthcare at $5,000 treatment

You've displayed **5 of 5 symptoms** of a new virus, with a **36% fatality rate**. **Treatment for the virus will cost you a total of $5,000**. How likely are you to see a healthcare professional?

0% chance of seeking healthcare at $5,000 treatment	5 of 5 symptoms, 36% fatality rate	100% chance of seeking healthcare at $5,000 treatment

1 of 5 symptoms 25000

You've displayed **1 of 5 symptoms** of a new virus, with a **1% fatality rate**. **Treatment for the virus will cost you a total of $25,000**. How likely are you to see a healthcare professional?

0% chance of seeking healthcare at $25,000 treatment	1 of 5 symptoms, 1% fatality rate	100% chance of seeking healthcare at $25,000 treatment

You've displayed **1 of 5 symptoms** of a new virus, with a **3% fatality rate**. **Treatment for the virus will cost you a total of $25,000**. How likely are you to see a healthcare professional?

0% chance of seeking healthcare at $25,000 treatment	1 of 5 symptoms, 3% fatality rate	100% chance of seeking healthcare at $25,000 treatment

You've displayed **1 of 5 symptoms** of a new virus, with a **9% fatality rate**. **Treatment for the virus will cost you a total of $25,000**. How likely are you to see a healthcare professional?

0% chance of seeking healthcare at $25,000	1 of 5 symptoms, 9% fatality rate	100% chance of seeking healthcare at $25,000

You've displayed **1 of 5 symptoms** of a new virus, with a **18% fatality rate**. **Treatment for the virus will cost you a total of $25,000**. How likely are you to see a healthcare professional?

0% chance of seeking healthcare at $25,000 treatment	1 of 5 symptoms, 18% fatality rate	100% chance of seeking healthcare at $25,000 treatment

You've displayed **1 of 5 symptoms** of a new virus, with a **36% fatality rate**. **Treatment for the virus will cost you a total of $25,000**. How likely are you to see a healthcare professional?

0% chance of seeking healthcare at $25,000 treatment	1 of 5 symptoms, 36% fatality rate	100% chance of seeking healthcare at $25,000 treatment

3 of 5 symptoms 25000

You've displayed **3 of 5 symptoms** of a new virus, with a **1% fatality rate**. **Treatment for the virus will cost you a total of $25,000**. How likely are you to see a healthcare professional?

0% chance of seeking healthcare at $25,000 treatment	3 of 5 symptoms, 1% fatality rate	100% chance of seeking healthcare at $25,000 treatment
	○	

You've displayed **3 of 5 symptoms** of a new virus, with a **3% fatality rate**. **Treatment for the virus will cost you a total of $25,000**. How likely are you to see a healthcare professional?

0% chance of seeking healthcare at $25,000 treatment	3 of 5 symptoms, 3% fatality rate	100% chance of seeking healthcare at $25,000 treatment
	○	

You've displayed **3 of 5 symptoms** of a new virus, with a **9% fatality rate**. **Treatment for the virus will cost you a total of $25,000**. How likely are you to see a healthcare professional?

0% chance of seeking healthcare at $25,000 treatment	3 of 5 symptoms, 9% fatality rate	100% chance of seeking healthcare at $25,000 treatment

You've displayed **3 of 5 symptoms** of a new virus, with a **18% fatality rate**. **Treatment for the virus will cost you a total of $25,000**. How likely are you to see a healthcare professional?

0% chance of seeking healthcare at $25,000 treatment	3 of 5 symptoms, 18% fatality rate	100% chance of seeking healthcare at $25,000 treatment

You've displayed **3 of 5 symptoms** of a new virus, with a **36% fatality rate**. **Treatment for the virus will cost you a total of $25,000**. How likely are you to see a healthcare professional?

0% chance of seeking healthcare at $25,000 treatment	3 of 5 symptoms, 36% fatality rate	100% chance of seeking healthcare at $25,000 treatment
	○	

5 of 5 symptoms 25000

You've displayed **5 of 5 symptoms** of a new virus, with a **1% fatality rate**. **Treatment for the virus will cost you a total of $25,000**. How likely are you to see a healthcare professional?

0% chance of seeking healthcare at $25,000 treatment	5 of 5 symptoms, 1% fatality rate	100% chance of seeking healthcare at $25,000 treatment
	○	

You've displayed **5 of 5 symptoms** of a new virus, with a **3% fatality rate**. **Treatment for the virus will cost you a total of $25,000**. How likely are you to see a healthcare professional?

0% chance of seeking healthcare at $25,000 treatment	5 of 5 symptoms, 3% fatality rate	100% chance of seeking healthcare at $25,000 treatment
	○	

You've displayed **5 of 5 symptoms** of a new virus, with a **9% fatality rate**. **Treatment for the virus will cost you a total of $25,000**. How likely are you to see a healthcare professional?

0% chance of seeking healthcare at $25,000 treatment	5 of 5 symptoms, 9% fatality rate	100% chance of seeking healthcare at $25,000 treatment
	○	

You've displayed **5 of 5 symptoms** of a new virus, with a **18% fatality rate**. **Treatment for the virus will cost you a total of $25,000**. How likely are you to see a healthcare professional?

0% chance of seeking healthcare at $25,000 treatment	5 of 5 symptoms, 18% fatality rate	100% chance of seeking healthcare at $25,000 treatment
	○	

You've displayed **5 of 5 symptoms** of a new virus, with a **36% fatality rate**. **Treatment for the virus will cost you a total of $25,000**. How likely are you to see a healthcare professional?

0% chance of seeking healthcare at $25,000 treatment	5 of 5 symptoms, 36% fatality rate	100% chance of seeking healthcare at $25,000 treatment
	○	

Attention Check

Would you rather have **$0 in a year** or have **$1000 immediately?**

$0 in a year $1000 immediately

Demographics break

You will now be asked a series of demographic, science, and health-related questions.

COVID General Questions

Please answer the following questions:

	Yes	No
Do you have an underlying health condition?	○	○
Do you have a family member with an underlying health condition?	○	○
Do you have any close friends with underlying health conditions?	○	○

Please describe the your, your family member's, or your friend's underlying health condition.

Please answer the following questions:

	Yes	No
Have you tested positive for COVID-19?	○	○
Has a family member of yours tested positive for COVID-19?	○	○
Has a close friend of yours tested positive for COVID-19?	○	○

Please describe the symptoms you, your family member, or your close friend experienced and the outcome.

Trust in Science McCright et al.

How much do you distrust or trust scientists to:

	Completely Distrust	Somewhat Distrust	Neither Trust or Distrust	Somewhat Trust	Completely Trust
Create knowledge that is unbiased and accurate?	○	○	○	○	○
Create knowledge that is useful?	○	○	○	○	○
Advise government officials on policy?	○	○	○	○	○
Inform the public on important issues?	○	○	○	○	○
	Completely Distrust	Somewhat Distrust	Neither Trust or Distrust	Somewhat Trust	Completely Trust

How much do you disagree or agree with the following statements:

	Completely Disagree	Somewhat Disagree	Neither Agree nor Disagree	Somewhat Agree	Completely Agree
Science is too concerned with theory and speculation to be of much use in making concrete government policy that will affect the way we live.	○	○	○	○	○
Science is too influenced by the politics of scientists to be of much use in making concrete government policy decisions that will affect the way we live.	○	○	○	○	○
Science is too influenced by government funding to be of much use in making concrete government policy decisions that will affect the way we live.	○	○	○	○	○
	Completely Disagree	Somewhat Disagree	Neither Agree nor Disagree	Somewhat Agree	Completely Agree

Many different scientists provide information that is used to inform government policy. For each of the following types of scientists, please tell us how much you distrust or trust them to advise elected officials on important science-based policy.

	Completely Distrust	Somewhat Distrust	Neither Trust nor Distrust	Somewhat Trust	Completely Trust
Food scientists who invent new processed food products in their laboratories.	○	○	○	○	○
Industrial chemists who create stronger synthetic materials for use in construction.	○	○	○	○	○
Oil geologists who identify new locations to drill for oil.	○	○	○	○	○
Agricultural scientists who create new fertilizers to boost agricultural production.	○	○	○	○	○

	Completely Distrust	Somewhat Distrust	Neither Trust nor Distrust	Somewhat Trust	Completely Trust
Materials scientists who help us design higher-quality screens for our smart phones.	○	○	○	○	○
Public health scientists who study the impacts of new types of processed food.	○	○	○	○	○
Epidemiologists who study the health risks of new synthetic materials used in housing construction.	○	○	○	○	○
Climate scientists who measure the amount of greenhouse gas in the atmosphere.	○	○	○	○	○

	Completely Distrust	Somewhat Distrust	Neither Trust nor Distrust	Somewhat Trust	Completely Trust
Wildlife ecologists who investigate how the disposal of human-made plastics affect wildlife habitats.	○	○	○	○	○

Oceanographers who research how pollution from agriculture is degrading coral reefs.	○	○	○	○	○
Environmental scientists who study the ecological impacts of mining for minerals used in smart phones.	○	○	○	○	○

Basic Demographics

The following questions are either fill in the blank or multiple choice. Please answer all these questions to the best of your abilities. All fields need to be filled to continue. If in a fill-in-the-blank option you are not sure, simply put NA or unsure in the text box.

Basic Demographics

Age

Gender Identity

Ethnic Identity

- ☐ Asian
- ☐ Black/African
- ☐ Caucasian
- ☐ Hispanic/Latin
- ☐ Pacific Islander
- ☐ ____________ Specify
- ☐ Prefer not to answer

What is the highest level of school you have completed or the highest degree you have received?

- ○ Less than high school degree
- ○ High school graduate (high school diploma or equivalent including GED)
- ○ Some college but no degree
- ○ Associate degree in college (2-year)
- ○ Bachelor's degree in college (4-year)
- ○ Master's degree
- ○ Doctoral degree
- ○ Professional degree (JD, MD)

Annual Income (approximate)

____

Politically, I consider myself to be

Very Conservative	Somewhat Conservative	Centrist	Somewhat Liberal	Very Liberal
		○		

How often do you floss?

At least once a day	at least once a week	at least once a month	at least once in six months	at least once a year	less than once a year
○	○	○	○	○	○

I would describe my general health to be

Very Poor	Somewhat Poor	Okay	Somewhat Good	Very Good

Health Insurance Status

- ○ Insured
- ○ Medicaid/Medicare
- ○ Uninsured
- ○ Family's/Spouse's Insurance

Comment Block

Please comment on your decision making processes when answering the hypothetical decision-making questions.

Please describe your thoughts regarding the COVID-19 (Coronavirus) pandemic.

completion break success

You have successfully completed the survey. The next page has your completion ID, please follow the instructions on that page to receive the completion ID and complete the survey. Prior to this, please complete the Captcha.

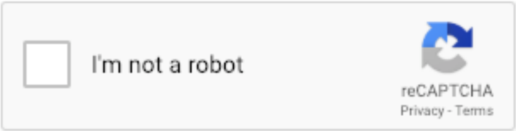

Completion ID Success

Your completion ID is ${e://Field/Success%20ID}, make sure you save this number as this indicates you have completed the survey.

Please paste this number ${e://Field/Success%20ID} into MTurk before pressing next . If you press next without saving the number, you may not be compensated for your participation.

After submitting this code into MTurk, you will be compensated the base of $0.25, and a bonus of $3.00 will awarded to you for completing the survey in it's entirety.

When you press next, you will have completed the survey.

Powered by Qualtrics
