# Supplementary material for: Delay discounting, probability discounting, and interdental cleaning frequency
Source: BMC Oral Health. 2022 Jul 29;22:315. doi: 10.1186/s12903-022-02328-6 (PMC9335449; doi:10.1186/s12903-022-02328-6)
Supplement: Supplementary file 2 — Additional file 2: Experimental instructions and question formats for Experiment 2. [file 12903_2022_2328_MOESM2_ESM.pdf]

Training Block

Prior to beginning the study, you will be required to complete 3 slider/percentage training questions, followed by a 4-question percentage comprehension test. If you do not pass the percentage comprehension test, you will be excluded from the study and ineligible for compensation. Please pay careful attention to the following examples and questions. This section should take less than 3 minutes. If you exit out of this survey, you will not be able to reopen it.

In the following questions you will be asked a series of questions involving ratios and the corresponding percentages associated with them. You will be using a slider bar to identify your answer. Understanding percentages is key to this study, as most questions will be in this format. Every answer will require to move or click the slider, even if it is already at the value you wish to choose. Below are some examples of how choosing between two options work in the context of this survey.

Selecting 100% A and 0% B

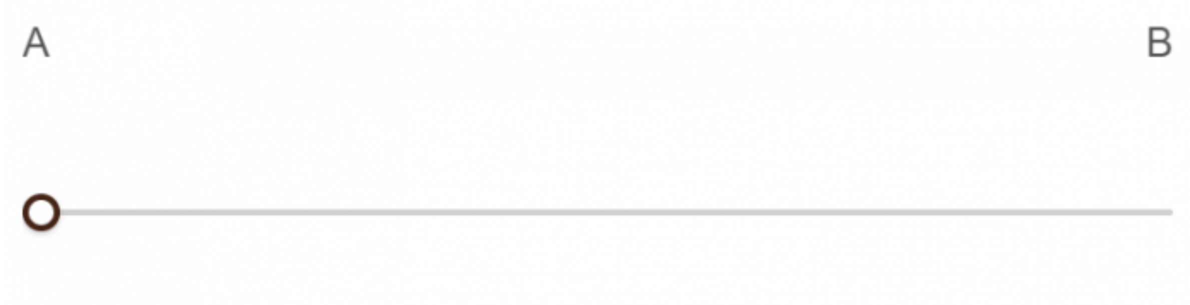

Selecting 75% A and 25% B

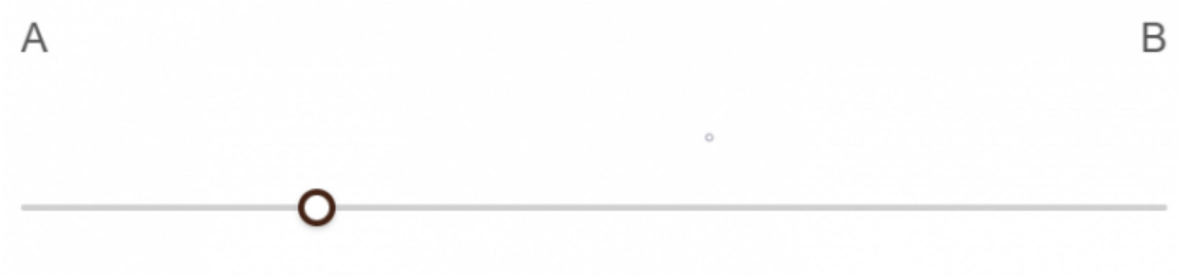

Selecting 50% A and 50% B

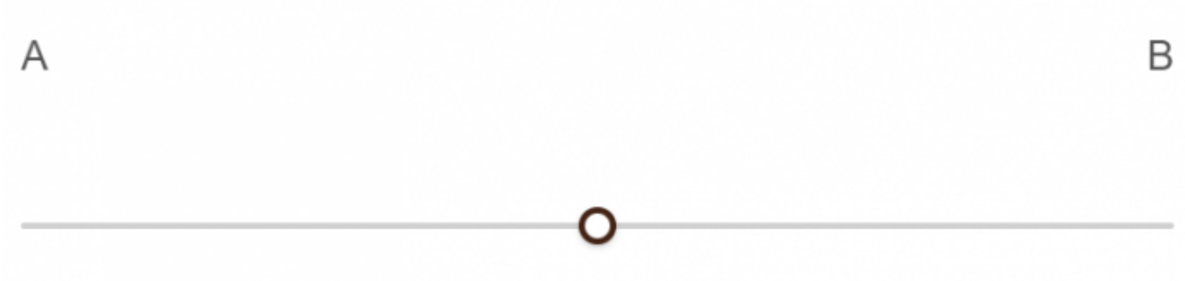

Selecting 75% B and 25% A

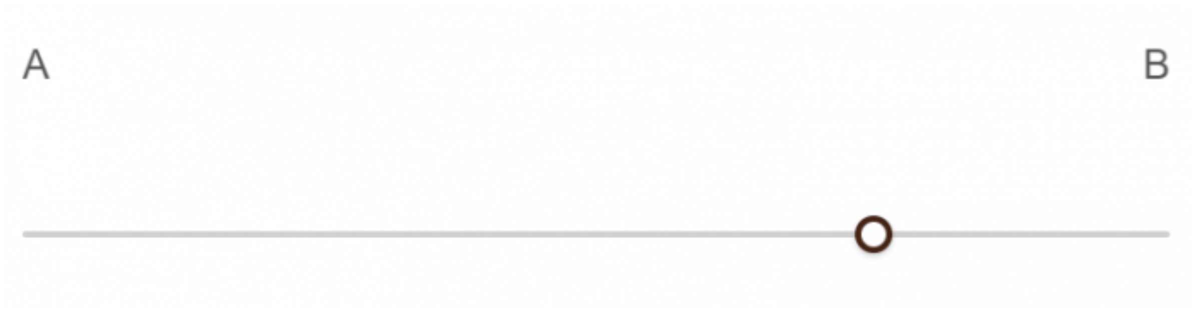

Selecting 100% B and 0% A

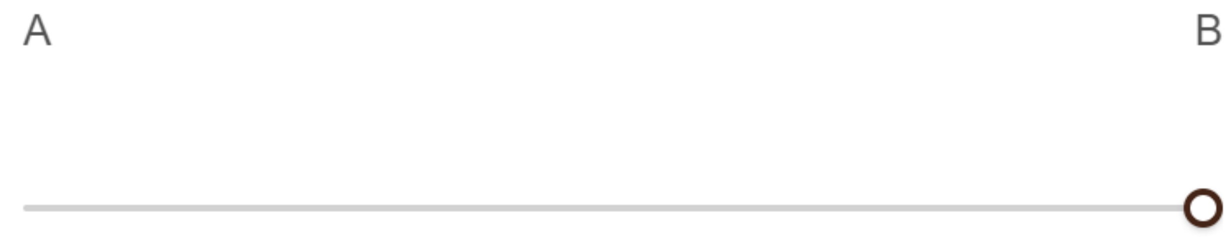

If you were to choose A 50% of the time and B 50% of the time, you would move the slider to the position seen below.

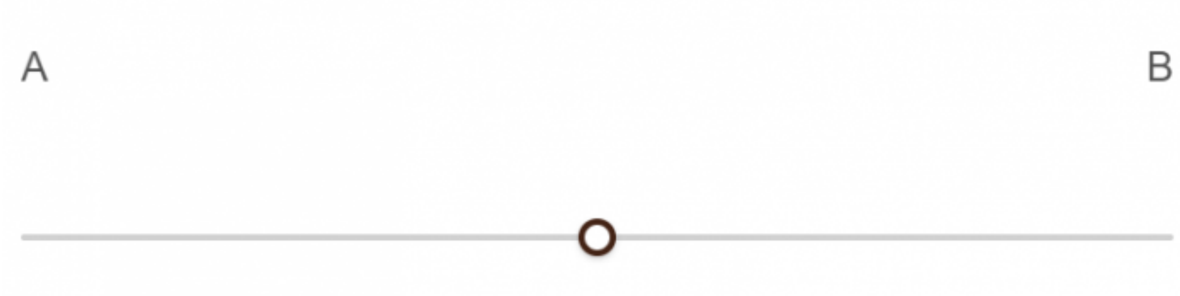

Please move the slider as if though you were choosing **<u>A 50%</u>** of the time and **<u>B 50%</u>** of the time

A B

0 25 50 75 100

If you were to choose A 100% of the time and B 0% of the time, you would move the slider to the leftmost position seen below.

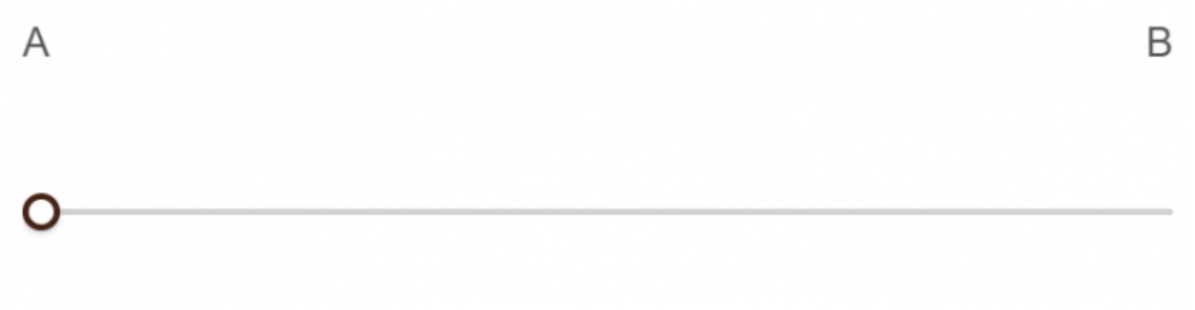

Please move the slider as if though you were choosing **<u>A 100%</u>** of the time and **<u>B 0%</u>** of the time

A B

0 25 50 75 100

If you were to choose B 75% of the time and A 25% of the time, you would move the slider to the position seen below.

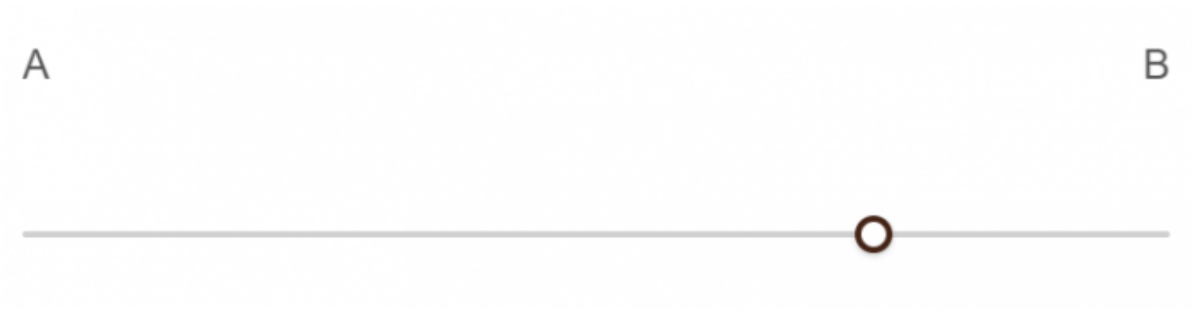

Please move the slider as if though you were choosing **B 75%** of the time and **A 25%** of the time

A B

0 25 50 75 100

You will now be tested on the previous content. Understanding percentages and the slider is necessary to continue on to the study. If you do not get all the four following questions correct you will be considered ineligible and removed from the study. You will only have one chance to answer these correctly, so please pay attention to the questions carefully. You will not get compensated if you are removed from the study in this way.

Testing Block

If you would **100%** choose **A** over B, move the slider to the correct spot

A B

0 25 50 75 100

If you would choose **A** **75%** of the time and **B** **25%** of the time, move the slider to the correct spot

A B

0 25 50 75 100

If you would choose **B** **50%** of the time and **A** **50%** of the time, move the slider to the correct spot

A B

0 25 50 75 100

If you would **100%** choose **B** over A, move the slider to the correct spot

A				B
0	25	50	75	100

Fail Screener

You did not pass the screener question.

When you press next, you will be exited from the survey.

Discounting Gain Questions Break

You have successfully completed the screener.

The following questions will consist of hypothetical choices between two monetary values that will vary in their likelihood and delay to getting the money. Answer these questions to the best of your ability and as if the situation were real. Note that there is no wrong way to answer these questions.

Delay Discounting Gain Small

What is the likelihood that you would select **gaining $500 immediately** over **gaining $1,000** in **1 day**?

Gain $500 immediately | Gain $1,000 in 1 day

What is the likelihood that you would select **gaining $500 immediately** over **gaining $1,000** in **1 month**?

Gain $500 immediately | Gain $1,000 in 1 month

What is the likelihood that you would select **gaining $500 immediately** over **gaining $1,000** in **6 months**?

Gain $500 immediately | Gain $1,000 in 6 months

What is the likelihood that you would select
gaining $500 immediately over **gaining $1,000** in **1 year**?

Gain $500 immediately | Gain $1,000 in 1 year

What is the likelihood that you would select
gaining $500 immediately over **gaining $1,000** in **2 years**?

Gain $500 immediately | Gain $1,000 in 2 years

Delay Discounting Loss Small

What is the likelihood that you would select
losing $500 immediately over **losing $1,000** in **1 day**?

Lose $500 immediately | Lose $1,000 in 1 day

What is the likelihood that you would select **losing $500 immediately** over **losing $1,000** in **1 month**?

Lose $500 immediately | Lose $1,000 in 1 month

What is the likelihood that you would select **losing $500 immediately** over **losing $1,000** in **6 months**?

Lose $500 immediately | Lose $1,000 in 6 months

What is the likelihood that you would select **losing $500 immediately** over **losing $1,000** in **1 year**?

Lose $500 immediately | Lose $1,000 in 1 year

What is the likelihood that you would select **losing $500 immediately** over **losing $1,000** in **2 years**?

Lose $500 immediately | Lose $1,000 in 2 years

Probability Discounting Gain Small

What is the likelihood that you would select a **100% chance of gaining $500** over a **99% chance of gaining $1,000**?

100% chance to gain $500 | 99% chance to gain $1,000

What is the likelihood that you would select a **100% chance of gaining $500** over an **80% chance of gaining $1,000**?

100% chance to gain $500 | 80% chance to gain $1,000

What is the likelihood that you would select a **100% chance of gaining $500** over a **50% chance of gaining $1,000**?

100% chance to gain $500 | 50% chance to gain $1,000

What is the likelihood that you would select a **100% chance of gaining $500** over a **20% chance of gaining $1,000**?

100% chance to gain $500 | 20% chance to gain $1,000

What is the likelihood that you would select a **100% chance of gaining $500** over a **1% chance of gaining $1,000**?

100% chance to gain $500 | 1% chance to gain $1,000

Probability Discounting Loss Small

What is the likelihood that you would select a **100% chance of losing $500** over a **99% chance of losing $1,000**?

100% chance to lose $500 | 99% chance to lose $1,000

What is the likelihood that you would select a **100% chance of losing $500** over an **80% chance of losing $1,000**?

100% chance to lose $500 | 80% chance to lose $1,000

What is the likelihood that you would select a **100% chance of losing $500** over a **50% chance of losing $1,000**?

100% chance to lose $500 | 50% chance to lose $1,000

What is the likelihood that you would select a **100% chance of losing $500** over a **20% chance of losing $1,000**?

100% chance to lose $500 20% chance to lose $1,000

What is the likelihood that you would select a **100% chance of losing $500** over a **1% chance of losing $1,000**?

100% chance to lose $500 1% chance to lose $1,000

Health Break

The following questions will ask you to imagine having experienced a particular symptom at a certain severity for some period of time with a given cost to access healthcare. **Assume that these symptoms are NOT related to COVID-19.** Answer the following questions as if the situation were real. Note that there is no wrong way to answer these questions.

Mild Headaches 10$

You have been experiencing **mild headaches** for the past **6 hours**. It will **cost you $10** to contact or see a healthcare professional. How likely are you to contact or see healthcare professional for your symptoms?

0% chance of contacting/seeing healthcare professional at $10	Mild headaches for 6 hours	100% chance of contacting/seeing healthcare professional at $10

You have been experiencing **mild headaches** for the past **day**. It will **cost you $10** to contact or see a healthcare professional. How likely are you to contact or see healthcare professional for your symptoms?

0% chance of contacting/seeing healthcare professional at $10	Mild headaches for 1 day	100% chance of contacting/seeing healthcare professional at $10

You have been experiencing **mild headaches** for the past **week**. It will **cost you $10** to contact or see a healthcare professional. How likely are you to contact or see healthcare professional for your symptoms?

0% chance of contacting/seeing healthcare professional at $10	Mild headaches for 1 week	100% chance of contacting/seeing healthcare professional at $10

You have been experiencing **mild headaches** for the past **month**. It will **cost you $10** to contact or see a healthcare professional. How likely are you to contact or see healthcare professional for your symptoms?

0% chance of contacting/seeing healthcare professional at $10	Mild headaches for 1 month	100% chance of contacting/seeing healthcare professional at $10

You have been experiencing **mild headaches** for the past **6 months**. It will **cost you $10** to contact or see a healthcare professional. How likely are you to contact or see healthcare professional for your symptoms?

0% chance of contacting/seeing healthcare professional at $10	Mild headaches for 6 months	100% chance of contacting/seeing healthcare professional at $10

Severe Headaches 10$

You have been experiencing **severe headaches** for the past **6 hours**. It will **cost you $10** to contact or see a healthcare professional. How likely are you to contact or see healthcare professional for your symptoms?

0% chance of contacting/seeing healthcare professional at $10	Severe headaches for 6 hours	100% chance of contacting/seeing healthcare professional at $10

You have been experiencing **severe headaches** for the past **day**. It will **cost you $10** to contact or see a healthcare professional. How likely are you to contact or see healthcare professional for your symptoms?

0% chance of contacting/seeing healthcare professional at $10	Severe headaches for 1 day	100% chance of contacting/seeing healthcare professional at $10

You have been experiencing **severe headaches** for the past **week**. It will **cost you $10** to contact or see a healthcare professional. How likely are you to contact or see healthcare professional for your symptoms?

0% chance of contacting/seeing healthcare professional at $10	Severe headaches for 1 week	100% chance of contacting/seeing healthcare professional at $10

You have been experiencing **severe headaches** for the past **month**. It will **cost you $10** to contact or see a healthcare professional. How likely are you to contact or see healthcare professional for your symptoms?

0% chance of contacting/seeing healthcare professional at $10	Severe headaches for 1 month	100% chance of contacting/seeing healthcare professional at $10

You have been experiencing **severe headaches** for the past **6 months**. It will **cost you $10** to contact or see a healthcare professional. How likely are you to contact or see healthcare professional for your symptoms?

0% chance of contacting/seeing healthcare professional at $10	Severe headaches for 6 months	100% chance of contacting/seeing healthcare professional at $10

Mild headaches 1000$

You have been experiencing **mild headaches** for the past **6 hours**. It will **cost you $1,000** to contact or see a healthcare professional. How likely are you to contact or see healthcare professional for your symptoms?

0% chance of contacting/seeing healthcare professional at $1,000	Mild headaches for 6 hours	100% chance of contacting/seeing healthcare professional at $1,000

You have been experiencing **mild headaches** for the past **day**. It will **cost you $1,000** to contact or see a healthcare professional. How likely are you to contact or see healthcare professional for your symptoms?

0% chance of contacting/seeing healthcare professional at $1,000	Mild headaches for 1 day	100% chance of contacting/seeing healthcare professional at $1,000

You have been experiencing **mild headaches** for the past **week**. It will **cost you $1,000** to contact or see a healthcare professional. How likely are you to contact or see healthcare professional for your symptoms?

0% chance of contacting/seeing healthcare professional at $1,000	Mild headaches for 1 week	100% chance of contacting/seeing healthcare professional at $1,000

You have been experiencing **mild headaches** for the past **month**. It will **cost you $1,000** to contact or see a healthcare professional. How likely are you to contact or see healthcare professional for your symptoms?

0% chance of contacting/seeing healthcare professional at $1,000	Mild headaches for 1 month	100% chance of contacting/seeing healthcare professional at $1,000

You have been experiencing **mild headaches** for the past **6 months**. It will **cost you $1,000** to contact or see a healthcare professional. How likely are you to contact or see healthcare professional for your symptoms?

0% chance of contacting/seeing healthcare professional at $1,000	Mild headaches for 6 months	100% chance of contacting/seeing healthcare professional at $1,000

Severe headaches $1000

You have been experiencing **severe headaches** for the past **6 hours**. It will **cost you $1,000** to contact or see a healthcare professional. How likely are you to contact or see healthcare professional for your symptoms?

0% chance of contacting/seeing healthcare professional at $1,000	Severe headaches for 6 hours	100% chance of contacting/seeing healthcare professional at $1,000

You have been experiencing **severe headaches** for the past **day**. It will **cost you $1,000** to contact or see a healthcare professional. How likely are you to contact or see healthcare professional for your symptoms?

0% chance of contacting/seeing healthcare professional at $1,000	Severe headaches for 1 day	100% chance of contacting/seeing healthcare professional at $1,000

You have been experiencing **severe headaches** for the past **week**. It will **cost you $1,000** to contact or see a healthcare professional. How likely are you to contact or see healthcare professional for your symptoms?

0% chance of contacting/seeing healthcare professional at $1,000	Severe headaches for 1 week	100% chance of contacting/seeing healthcare professional at $1,000

You have been experiencing **severe headaches** for the past **month**. It will **cost you $1,000** to contact or see a healthcare professional. How likely are you to contact or see healthcare professional for your symptoms?

0% chance of contacting/seeing healthcare professional at $1,000	Severe headaches for 1 month	100% chance of contacting/seeing healthcare professional at $1,000

You have been experiencing **severe headaches** for the past **6 months**. It will **cost you $1,000** to contact or see a healthcare professional. How likely are you to contact or see healthcare professional for your symptoms?

0% chance of contacting/seeing healthcare professional at $1,000	Severe headaches for 6 months	100% chance of contacting/seeing healthcare professional at $1,000

Mild cough $10

You have been experiencing a **mild cough** for the past **6 hours**. It will **cost you $10** to contact or see a healthcare professional. How likely are you to contact or see healthcare professional for your symptoms?

0% chance of contacting/seeing healthcare professional at $10	Mild cough for 6 hours	100% chance of contacting/seeing healthcare professional at $10

You have been experiencing a **mild cough** for the past **day**. It will **cost you $10** to contact or see a healthcare professional. How likely are you to contact or see healthcare professional for your symptoms?

0% chance of contacting/seeing healthcare professional at $10	Mild cough for 1 day	100% chance of contacting/seeing healthcare professional at $10

You have been experiencing a **mild cough** for the past **week**. It will **cost you $10** to contact or see a healthcare professional. How likely are you to contact or see healthcare professional for your symptoms?

0% chance of contacting/seeing healthcare professional at $10	Mild cough for 1 week	100% chance of contacting/seeing healthcare professional at $10

You have been experiencing a **mild cough** for the past **month**. It will **cost you $10** to contact or see a healthcare professional. How likely are you to contact or see healthcare professional for your symptoms?

0% chance of contacting/seeing healthcare professional at $10	Mild cough for 1 month	100% chance of contacting/seeing healthcare professional at $10

You have been experiencing a **mild cough** for the past **6 months**. It will **cost you $10** to contact or see a healthcare professional. How likely are you to contact or see healthcare professional for your symptoms?

0% chance of contacting/seeing healthcare professional at $10		100% chance of contacting/seeing healthcare professional at $10
	Mild cough for 6 months	

severe cough $10

You have been experiencing a **severe cough** for the past **6 hours**. It will **cost you $10** to contact or see a healthcare professional. How likely are you to contact or see healthcare professional for your symptoms?

0% chance of contacting/seeing healthcare professional at $10		100% chance of contacting/seeing healthcare professional at $10
	Severe cough for 6 hours	

You have been experiencing a **severe cough** for the past **day**. It will **cost you $10** to contact or see a healthcare professional. How likely are you to contact or see healthcare professional for your symptoms?

0% chance of contacting/seeing healthcare professional at $10	Severe cough for 1 day	100% chance of contacting/seeing healthcare professional at $10

You have been experiencing a **severe cough** for the past **week**. It will **cost you $10** to contact or see a healthcare professional. How likely are you to contact or see healthcare professional for your symptoms?

0% chance of contacting/seeing healthcare professional at $10	Severe cough for 1 week	100% chance of contacting/seeing healthcare professional at $10

You have been experiencing a **severe cough** for the past **month**. It will **cost you $10** to contact or see a healthcare professional. How likely are you to contact or see healthcare professional for your symptoms?

0% chance of contacting/seeing healthcare professional at $10	Severe cough for 1 month	100% chance of contacting/seeing healthcare professional at $10

You have been experiencing a **severe cough** for the past **6 months**. It will **cost you $10** to contact or see a healthcare professional. How likely are you to contact or see healthcare professional for your symptoms?

0% chance of contacting/seeing healthcare professional at $10	Severe cough for 6 months	100% chance of contacting/seeing healthcare professional at $10

mild cough $1000

You have been experiencing a **mild cough** for the past **6 hours**. It will **cost you $1,000** to contact or see a healthcare professional. How likely are you to contact or see healthcare professional for your symptoms?

0% chance of contacting/seeing healthcare professional at $1,000	Mild cough for 6 hours	100% chance of contacting/seeing healthcare professional at $1,000

You have been experiencing a **mild cough** for the past **day**. It will **cost you $1,000** to contact or see a healthcare professional. How likely are you to contact or see healthcare professional for your symptoms?

0% chance of contacting/seeing healthcare professional at $1,000	Mild cough for 1 day	100% chance of contacting/seeing healthcare professional at $1,000

You have been experiencing a **mild cough** for the past **week**. It will **cost you $1,000** to contact or see a healthcare professional. How likely are you to contact or see healthcare professional for your symptoms?

0% chance of contacting/seeing healthcare professional at $1,000	Mild cough for 1 week	100% chance of contacting/seeing healthcare professional at $1,000

You have been experiencing a **mild cough** for the past **month**. It will **cost you $1,000** to contact or see a healthcare professional. How likely are you to contact or see healthcare professional for your symptoms?

0% chance of contacting/seeing healthcare professional at $1,000	Mild cough for 1 month	100% chance of contacting/seeing healthcare professional at $1,000

You have been experiencing a **mild cough** for the past **6 months**. It will **cost you $1,000** to contact or see a healthcare professional. How likely are you to contact or see healthcare professional for your symptoms?

0% chance of contacting/seeing healthcare professional at $1,000		100% chance of contacting/seeing healthcare professional at $1,000
	Mild cough for 6 months	

severe cough $1000

You have been experiencing a **severe cough** for the past **6 hours**. It will **cost you $1,000** to contact or see a healthcare professional. How likely are you to contact or see healthcare professional for your symptoms?

0% chance of contacting/seeing healthcare professional at $1,000		100% chance of contacting/seeing healthcare professional at $1,000
	Severe cough for 6 hours	

You have been experiencing a **severe cough** for the past **day**. It will **cost you $1,000** to contact or see a healthcare professional. How likely are you to contact or see healthcare professional for your symptoms?

0% chance of contacting/seeing healthcare professional at $1,000	Severe cough for 1 day	100% chance of contacting/seeing healthcare professional at $1,000

You have been experiencing a **severe cough** for the past **week**. It will **cost you $1,000** to contact or see a healthcare professional. How likely are you to contact or see healthcare professional for your symptoms?

0% chance of contacting/seeing healthcare professional at $1,000	Severe cough for 1 week	100% chance of contacting/seeing healthcare professional at $1,000

You have been experiencing a **severe cough** for the past **month**. It will **cost you $1,000** to contact or see a healthcare professional. How likely are you to contact or see healthcare professional for your symptoms?

0% chance of contacting/seeing healthcare professional at $1,000	Severe cough for 1 month	100% chance of contacting/seeing healthcare professional at $1,000

You have been experiencing a **severe cough** for the past **6 months**. It will **cost you $1,000** to contact or see a healthcare professional. How likely are you to contact or see healthcare professional for your symptoms?

0% chance of contacting/seeing healthcare professional at $1,000	Severe cough for 6 months	100% chance of contacting/seeing healthcare professional at $1,000

Mild nausea 10$

You have been experiencing **mild nausea** for the past **6 hours**. It will **cost you $10** to contact or see a healthcare professional. How likely are you to contact or see healthcare professional for your symptoms?

0% chance of contacting/seeing healthcare professional at $10	Mild nausea for 6 hours	100% chance of contacting/seeing healthcare professional at $10

You have been experiencing **mild nausea** for the past **day**. It will **cost you $10** to contact or see a healthcare professional. How likely are you to contact or see healthcare professional for your symptoms?

0% chance of contacting/seeing healthcare professional at $10	Mild nausea for 1 day	100% chance of contacting/seeing healthcare professional at $10

You have been experiencing **mild nausea** for the past **week**. It will **cost you $10** to contact or see a healthcare professional. How likely are you to contact or see healthcare professional for your symptoms?

0% chance of contacting/seeing healthcare professional at $10	Mild nausea for 1 week	100% chance of contacting/seeing healthcare professional at $10

You have been experiencing **mild nausea** for the past **month**. It will **cost you $10** to contact or see a healthcare professional. How likely are you to contact or see healthcare professional for your symptoms?

0% chance of contacting/seeing healthcare professional at $10	Mild nausea for 1 month	100% chance of contacting/seeing healthcare professional at $10

You have been experiencing **mild nausea** for the past **6 months**. It will **cost you $10** to contact or see a healthcare professional. How likely are you to contact or see healthcare professional for your symptoms?

0% chance of contacting/seeing healthcare professional at $10	Mild nausea for 6 months	100% chance of contacting/seeing healthcare professional at $10

Severe Nausea $10

You have been experiencing **severe nausea** for the past **6 hours**. It will **cost you $10** to contact or see a healthcare professional. How likely are you to contact or see healthcare professional for your symptoms?

0% chance of contacting/seeing healthcare professional at $10	Severe nausea for 6 hours	100% chance of contacting/seeing healthcare professional at $10

You have been experiencing **severe nausea** for the past **day**. It will **cost you $10** to contact or see a healthcare professional. How likely are you to contact or see healthcare professional for your symptoms?

0% chance of contacting/seeing healthcare professional at $10	Severe nausea for 1 day	100% chance of contacting/seeing healthcare professional at $10

You have been experiencing **severe nausea** for the past **week**. It will **cost you $10** to contact or see a healthcare professional. How likely are you to contact or see healthcare professional for your symptoms?

0% chance of contacting/seeing healthcare professional at $10	Severe nausea for 1 week	100% chance of contacting/seeing healthcare professional at $10

You have been experiencing **severe nausea** for the past **month**. It will **cost you $10** to contact or see a healthcare professional. How likely are you to contact or see healthcare professional for your symptoms?

0% chance of contacting/seeing healthcare professional at $10	Severe nausea for 1 month	100% chance of contacting/seeing healthcare professional at $10

You have been experiencing **severe nausea** for the past **6 months**. It will **cost you $10** to contact or see a healthcare professional. How likely are you to contact or see healthcare professional for your symptoms?

0% chance of contacting/seeing healthcare professional at $10	Severe nausea for 6 months	100% chance of contacting/seeing healthcare professional at $10

mild nausea $1000

You have been experiencing **mild nausea** for the past **6 hours**. It will **cost you $1,000** to contact or see a healthcare professional. How likely are you to contact or see healthcare professional for your symptoms?

0% chance of contacting/seeing healthcare professional at $1,000	Mild nausea for 6 hours	100% chance of contacting/seeing healthcare professional at $1,000

You have been experiencing **mild nausea** for the past **day**. It will **cost you $1,000** to contact or see a healthcare professional. How likely are you to contact or see healthcare professional for your symptoms?

0% chance of contacting/seeing healthcare professional at $1,000	Mild nausea for 1 day	100% chance of contacting/seeing healthcare professional at $1,000

You have been experiencing **mild nausea** for the past **week**. It will **cost you $1,000** to contact or see a healthcare professional. How likely are you to contact or see healthcare professional for your symptoms?

0% chance of contacting/seeing healthcare professional at $1,000	Mild nausea for 1 week	100% chance of contacting/seeing healthcare professional at $1,000

You have been experiencing **mild nausea** for the past **month**. It will **cost you $1,000** to contact or see a healthcare professional. How likely are you to contact or see healthcare professional for your symptoms?

0% chance of contacting/seeing healthcare professional at $1,000	Mild nausea for 1 month	100% chance of contacting/seeing healthcare professional at $1,000

You have been experiencing **mild nausea** for the past **6 months**. It will **cost you $1,000** to contact or see a healthcare professional. How likely are you to contact or see healthcare professional for your symptoms?

0% chance of contacting/seeing healthcare professional at $1,000	Mild nausea for 6 months	100% chance of contacting/seeing healthcare professional at $1,000

Severe nausea $1000

You have been experiencing **severe nausea** for the past **6 hours**. It will **cost you $1,000** to contact or see a healthcare professional. How likely are you to contact or see healthcare professional for your symptoms?

0% chance of contacting/seeing healthcare professional at $1,000	Severe nausea for 6 hours	100% chance of contacting/seeing healthcare professional at $1,000

You have been experiencing **severe nausea** for the past **day**. It will **cost you $1,000** to contact or see a healthcare professional. How likely are you to contact or see healthcare professional for your symptoms?

0% chance of contacting/seeing healthcare professional at $1,000	Severe nausea for 1 day	100% chance of contacting/seeing healthcare professional at $1,000

You have been experiencing **severe nausea** for the past **week**. It will **cost you $1,000** to contact or see a healthcare professional. How likely are you to contact or see healthcare professional for your symptoms?

0% chance of contacting/seeing healthcare professional at $1,000	Severe nausea for 1 week	100% chance of contacting/seeing healthcare professional at $1,000

You have been experiencing **severe nausea** for the past **month**. It will **cost you $1,000** to contact or see a healthcare professional. How likely are you to contact or see healthcare professional for your symptoms?

0% chance of contacting/seeing healthcare professional at $1,000	Severe nausea for 1 month	100% chance of contacting/seeing healthcare professional at $1,000

You have been experiencing **severe nausea** for the past **6 months**. It will **cost you $1,000** to contact or see a healthcare professional. How likely are you to contact or see healthcare professional for your symptoms?

0% chance of contacting/seeing healthcare professional at $1,000	Severe nausea for 6 months	100% chance of contacting/seeing healthcare professional at $1,000

Attention Check

Would you rather have **$1000 immediately** or have **$1 in a year?**

$1000 immediately $1 in a year

Demographics break

You will now be asked a series of demographic and health-related questions.

Symptom Concerns: All

With regard to nausea, what is your overall concern about experiencing...

	Not at all concerned			Somewhat Concerned			Very Concerned	
	0	1	2	3	4	5	6	
Mild Nausea								
Moderate Nausea								
Severe Nausea								

With regard to headaches, what is your overall concern about experiencing...

	Not at all concerned			Somewhat Concerned			Very Concerned	
	0	1	2	3	4	5	6	
Mild Headaches								
Moderate Headaches								
Severe Headaches								

With regard to coughing, what is you overall concern about experiencing...

	Not at all concerned			Somewhat Concerned			Very Concerned	
	0	1	2	3	4	5	6	
Mild Coughing								
Moderate Coughing								
Severe Coughing								

Basic Demographics

The following questions are either fill in the blank or multiple choice.

Please answer all these questions to the best of your abilities. All fields need to be filled to continue. If in a fill-in-the-blank option you are not sure, simply put NA or unsure in the text box.

Basic Demographics

Age ______

Gender Identity ______

Ethnic Identity

- ☐ Asian
- ☐ Black/African
- ☐ Caucasian
- ☐ Hispanic/Latin
- ☐ Pacific Islander
- ☐ ______ Specify
- ☐ Prefer not to answer

What is the highest level of school you have completed or the highest degree you have received?

- Less than high school degree
- High school graduate (high school diploma or equivalent including GED)
- Some college but no degree
- Associate degree in college (2-year)
- Bachelor's degree in college (4-year)
- Master's degree
- Doctoral degree
- Professional degree (JD, MD)

Annual Income (approximate)

Do you have employment other than MTurk?

- Yes
- No

Did you lose your job due to COVID-19?

- Yes
- No

What is your other employment?

How often do you floss?

At least once a day	at least once a week	at least once a month	at least once in six months	at least once a year	less than once a year
○	○	○	○	○	○

Health Insurance Status

- ○ Insured
- ○ Medicaid/Medicare
- ○ Uninsured
- ○ Family's/Spouse's Insurance

Amount insured

Percent covered by insurance

Deductible

Did you lose health insurance due to COVID-19 related reasons (e.g., job loss, budget cuts, inability to pay for health insurance)?

- ◯ Yes
- ◯ No

Please describe the reason you lost your health insurance due to COVID-19

Trust In Doctors

Please answer the following question regarding medical doctors.

	Strongly Disagree	Disagree	Neutral	Agree	Strongly Agree
Sometimes doctors care more about what is convenient for them that about their patient's medical needs.	○	○	○	○	○
Doctors are extremely thorough and helpful.	○	○	○	○	○
You completely trust doctors' decisions about which medical treatments are best.	○	○	○	○	○
A doctor would never mislead you about anything	○	○	○	○	○
All in all, you trust doctors completely	○	○	○	○	○
	Strongly Disagree	Disagree	Neutral	Agree	Strongly Agree

Fagerstrom Nicotine

Do you smoke cigarettes?

- ○ Yes
- ○ No

How soon after you wake up do you smoke your first cigarette?

- ○ Within 5 minutes
- ○ 6 - 30 minutes
- ○ 31 - 60 minutes
- ○ After 60 minutes

Do you find it difficult to refrain from smoking in places where it is forbidden?

- Yes
- No

Which cigarette would you hate most to give up?

- The first one in the morning
- All others

How many cigarettes a day do you smoke?

- 10 or less
- 11 - 20
- 21 - 30
- 31 or more

Do you smoke more frequently during the first hours after waking than during the rest of the day?

- Yes
- No

Do you smoke more frequently during the first hours after waking than during the rest of the day?

- ◯ Yes
- ◯ No

Do you smoke if you are so ill that you are in bed most of the day?

- ◯ Yes
- ◯ No

SF-12

In general , would you say your health is:

- ◯ Excellent
- ◯ Very good
- ◯ Good
- ◯ Fair
- ◯ Poor

The following items are about activities you might do during a typical day, does your health now limit you in these activities? If so, how much?

	Yes, limited a lot	Yes, limited a little	No, not limited at all
Moderate activities, such as moving a table, pushing a vacuum cleaner, bowling, or playing golf.	○	○	○
Climbing several flights of stairs	○	○	○

During the past 4 weeks, have you had any of the following problems with your work or other regular daily activities as a result of your physical health?

	Yes	No
Accomplished less than you would like	○	○
Were limited in the kind of work or activities	○	○

During the past 4 weeks, have you had any of the following problems with your work or other regular daily activities as a result of any emotional problems (such as feeling depressed or anxious)?

	Yes	No
Accomplished less than you would like	○	○
Didn’t do work or other activities as carefully as usual	○	○

During the past 4 weeks, how much did pain interfere with your normal work (including both work outside the home and housework)?

- Not at all
- A little bit
- Moderately
- Quite a bit
- Extremely

These questions are about how you feel and how things have been with you during the past 4 weeks. For each questions, please give the answer that comes closest to the way you have been feeling. How much time in the past 4 weeks .

	All of the time	Most of the time	A good bit of the time	Some of the time	A little bit of the time	None of the time
Have you felt calm and peaceful?	○	○	○	○	○	○
Did you have a lot of energy?	○	○	○	○	○	○
Have you felt downhearted and blue?	○	○	○	○	○	○

During the past 4 weeks, how much of the time has your physical health or emotional problems interfered with your social activities (like visiting with friends, relatives, etc.)?

- ◯ All of the time
- ◯ Most of the time
- ◯ Some of the time
- ◯ A little of the time
- ◯ None of the time

Underlying conditions and insurance questions

Please answer the following questions:

	Yes	No
Have you ever avoided going to a doctor for an illness due to cost?	◯	◯
Have you ever delayed going to a doctor for an illness due to cost?	◯	◯

Please describe in more detail your experience of delaying/refusing /avoiding some medical procedure/visit due to cost.

Please answer the following questions:

	Yes	No
Do you have an underlying health condition?	○	○
Do you have a family member with an underlying health condition?	○	○
Do you have any close friends with underlying health conditions?	○	○

Please describe your, your family member's, or your friend's underlying health condition.

Comment Block

Please comment on your decision making processes when answering the hypothetical decision-making questions.

completion break success

You have successfully completed the survey. The next page has your completion ID, please follow the instructions on that page to receive the completion ID and complete the survey. Prior to this, please complete the Captcha.

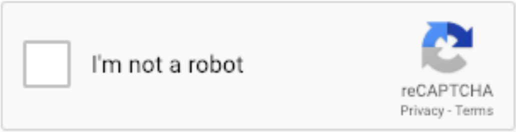

Completion ID Success

Your completion ID is ${e://Field/Success%20ID}, make sure you save this number as this indicates you have completed the survey.

Please paste this number ${e://Field/Success%20ID} into MTurk before pressing next . If you press next without saving the number, you may not be compensated for your participation.

After submitting this code into MTurk, you will be compensated $3.50 for completing the survey in it's entirety.

When you press next, you will have completed the survey.

WESTERN MICHIGAN UNIVERSITY

Powered by Qualtrics
